# DNA damage alters EGFR signaling and reprograms cellular response via Mre-11

**DOI:** 10.1038/s41598-022-09779-5

**Published:** 2022-04-06

**Authors:** Yael Volman, Ruth Hefetz, Eithan Galun, Jacob Rachmilewitz

**Affiliations:** Goldyne Savad Institute of Gene Therapy, Hadassah Medical Center, Faculty of Medicine, Hebrew University of Jerusalem, P.O.B. 12000, 91120 Jerusalem, Israel

**Keywords:** Cell biology, Molecular biology

## Abstract

To combat the various DNA lesions and their harmful effects, cells have evolved different strategies, collectively referred as DNA damage response (DDR). The DDR largely relies on intranuclear protein networks, which sense DNA lesions, recruit DNA repair enzymes, and coordinates several aspects of the cellular response, including a temporary cell cycle arrest. In addition, external cues mediated by the surface EGF receptor (EGFR) through downstream signaling pathways contribute to the cellular DNA repair capacity. However, cell cycle progression driven by EGFR activation should be reconciled with cell cycle arrest necessary for effective DNA repair. Here, we show that in damaged cells, the expression of Mig-6 (mitogen-inducible gene 6), a known regulator of EGFR signaling, is reduced resulting in heightened EGFR phosphorylation and downstream signaling. These changes in Mig-6 expression and EGFR signaling do not occur in cells deficient of Mre-11, a component of the MRN complex, playing a central role in double-strand break (DSB) repair or when cells are treated with the MRN inhibitor, mirin. RNAseq and functional analysis reveal that DNA damage induces a shift in cell response to EGFR triggering that potentiates DDR-induced p53 pathway and cell cycle arrest. These data demonstrate that the cellular response to EGFR triggering is skewed by components of the DDR, thus providing a plausible explanation for the paradox of the known role played by a growth factor such as EGFR in the DNA damage repair.

## Introduction

Cells are continually exposed to DNA damaging agents of endogenous (metabolic) and environmental origin. Double-strand breaks (DSBs) threaten chromosomal integrity and must be repaired efficiently to maintain cell viability and to prevent malignant transformation. Diverse and complex molecular mechanisms, collectively termed the DNA damage response (DDR), have evolved to alleviate the consequences of genotoxic damage and to maintain tissue homeostasis. The DDR largely relies on intranuclear protein networks, which sense DNA lesions and recruit DNA repair enzymes.

The Mre-11–Rad50–Nbs1 (MRN) and the Ku heterodimer (Ku70-Ku80) complexes are among the ‘first responders’ that bind to DSBs independently of other factors. These complexes play important roles in sensing and signaling as well as promoting the repair of DNA breaks via both homologous recombination (HR) and non-homologous end-joining (NHEJ)^1–5^. MRN and Ku70/80 complexes play key roles in recruiting and activating the kinases ataxia-telangiectasia mutated (ATM) and DNA-dependent protein kinase catalytic subunit (DNA-PKcs), respectively^6–9^. In turn, these phosphoinositide-3-kinase (PI3K) family members phosphorylate other proteins including the histone H2AX (referred to as γH2AX) over a large region surrounding the DSB, leading to readily visualized foci, and creating binding sites for other DDR proteins^5,10^.

While DDR is essentially a cell-autonomous process that occurs in the nucleus, the cell surface epidermal growth factor receptor (EGFR) has been shown to accelerate DSB rejoining. The EGFR itself (upon nuclear translocation) or by signaling through ERK and AKT, regulates the phosphorylation, distribution, and activity of key proteins in the DDR pathway (reviewed in^11^). We recently demonstrated that macrophages, via the release of heparin-binding EGF-like growth factor (HB-EGF), facilitate DSBs repair in EGFR expressing neighboring cells^12^, consistent with the notion that DNA damage is controlled, in part, by extracellular cues.

The EGFR belongs to the ErbB family of receptor tyrosine kinases (RTKs), exerts critical functions in cell physiology, and is frequently mutated and/or overexpressed in human cancer^13–15^.

Deregulated EGFR signaling in cancer has been linked to resistance to chemo- and/or radiotherapy as well as increased DNA damage repair capacity, whereas inhibition of the EGFR attenuates DNA damage repair^16–19^. Of course, beyond effects related to DNA repair, ErbB family members including the EGFR itself are master regulators of cell cycle progression and survival in multiple cell types including fibroblasts and epithelial cells^14,15,20^.

While numerous reports have demonstrated that EGFR signaling is playing a role in DNA damage response, little is known how the diverse effects of EGFR activation are coordinated in cells challenged by DNA damaging agents. Specifically, it is reasonable to assume that cell-cycle progression driven by EGFR activation may counteract the cell-cycle arrest necessary for effective DNA repair to occur. We hypothesize that DNA damage modifies signal transduction triggered by EGFR activation. We further suggest that these ‘inside-out’ signaling events serve an essential role in synchronizing cell-cycle progression and cell survival mechanisms with DDR to optimize conditions for cell recovery and DNA damage repair.

## Results

### DNA damage enhances ligand-dependent EGFR-induced signaling

**Figure 1:**
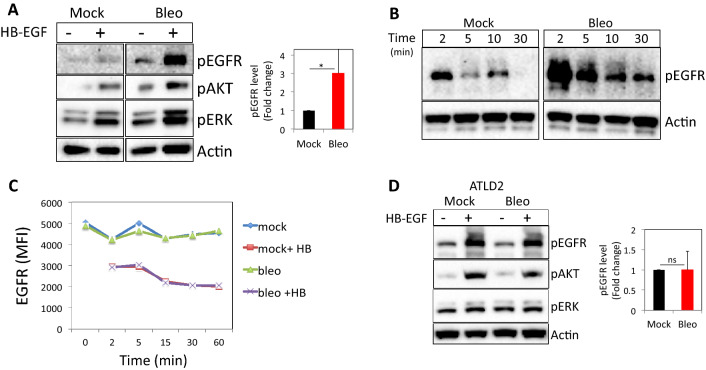
DNA damage enhances ligand-induced EGFR phosphorylation and signaling. Normal human dermal fibroblasts (HF) were either left untreated or treated with bleomycin for 30 min, followed by extensive washing and subsequent exposure to HB-EGF (10 ng/ml). After 5 min (**A**) or at indicated time points (**B**), cells were lysed. Western blot analyses were performed on cell extracts using antibodies to phosphorylated EGFR (Y1173), phosphorylated AKT, and ERK. Anti-β-actin immunoblotting revealed relative amounts of protein in each lane. (**C**) HF were treated as above and at the indicated time points cells were collected and immune-stained for cell surface expression of EGFR. MFI: Median fluorescent intensity. (**D**) Fibroblasts from an ATLD patient (ATLD2) were treated and analyzed as in (**A**). pEGFR signal intensities were quantified and normalized to actin signal (loading control). The level of EGFR phosphorylation in response to HB-EGF treatment in bleomycin treated cells as compared to that of mock-treated cells (set as 1) are shown (**A**: n = 9; **D**: n = 4). Representative results of at least three independent experiments are shown. *p < 0.05; ns: non-significant.

To determine whether DNA damage regulates EGFR signaling, we monitored HB-EGF-induced EGFR-signaling events in cells after bleomycin treatment (Fig. S1C and^21,22^) and compared them to HB-EGF-treated controls. Normal human dermal fibroblasts (HF) pretreated with bleomycin and subsequently exposed to HB-EGF exhibited higher levels of pEGFR (Y1173; on average threefold higher, n = 9), pAKT and pERK signals when compared to cells treated with HB-EGF alone (Fig. 1A). Similar results were obtained when DNA damage was induced by peroxide (Fig. S1A,B). Moreover, HB-EGF treatment of mock-treated fibroblasts induced short-term EGFR phosphorylation, which returned to baseline levels within 10 min, whereas fibroblasts pretreated with bleomycin and subsequently exposed to HB-EGF exhibited higher levels of pEGFR for an extended time of up to 30 min (Fig. 1B).

Using an EGFR phosphorylation antibody array, we simultaneously assessed the relative levels of phosphorylation of 17 different sites for the human EGFR family^23^. The preferential phosphorylated site upon HB-EGF stimulation was Y845. Moreover, ErbB2Y1112 and ErbB2Ser113 were also phosphorylated upon HB-EGF treatment, but to a lesser extent in bleomycin-treated cells. On the other hand, HB-EGF-induced phosphorylation of site Y1173 was ~ threefold higher in bleomycin-treated cells as compared to control cells, verifying the above findings (Fig. S1C).

Activation of cell surface receptors triggers regulatory processes that restrict the duration of signaling via ligand-induced internalization. One possible explanation for EGFR enhanced and prolonged stimulation in DNA damaged cells might be a higher number of surface receptors, partially due to incomplete ligand-induced internalization. To test this possibility, we monitored EGFR surface expression in fibroblasts that were either left as control or treated with bleomycin at various time points after HB-EGF incubation. Of note, as shown in Fig. 1C, there was no significant difference in the level of cell surface EGFR in control vs bleomycin-treated cells with a similar extent of HB-EGF-induced receptor downregulation (Fig. 1C).

Next, we used fibroblasts from a patient with ataxia telangiectasia-like disorder (ATLD), a human syndrome with hypomorphic mutations in Mre-11^24^. This cell line (kindly provided by Y. Shiloh from Tel Aviv University) was used to determine whether the DNA damage-associated increase in EGFR activation is dependent on sensing DNA damage by the MRN pathway. ATLD2 cells responded normally to HB-EGF with increased pEGFR, pAKT, and pERK. However, in contrast to wild-type HF (Fig. 1A), in ATLD2 cells treated with bleomycin, the level of HB-EGF induced EGFR, AKT, and ERK phosphorylations were not elevated (Fig. 1D). These results suggest that Mre-11 is required for increased EGFR activation by exogenous ligands in the context of DNA damage.

### Pharmacological inhibition of Mre-11 inhibits EGFR activation only in cells suffering from DNA damage

**Figure 2:**
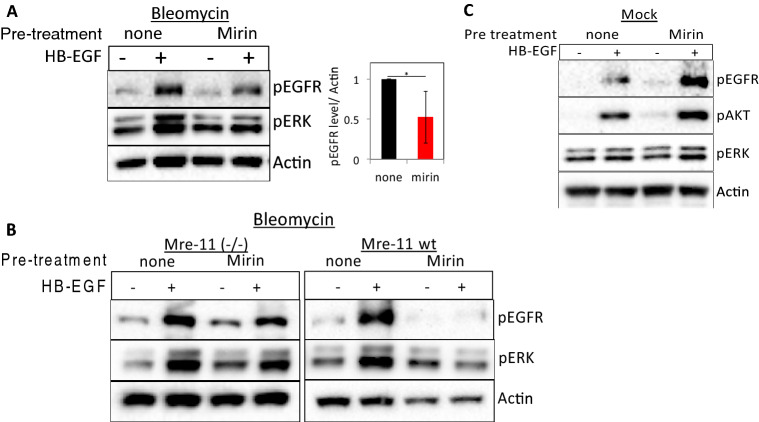
Pharmacological inhibition of Mre-11 interferes with ligand-dependent EGFR signaling in cells suffering from DNA damage. (**A**) HF were either left untreated or treated with mirin (100 µM) for one hour and then were treated with bleomycin for an additional 30 min followed by HB-EGF (10 ng/ml) treatment for 10 min. (**B**) ATLD2 cells (Mre-11^−/−^) or ATLD2 cells reconstituted with wild type Mre-11 (Mre-11 wt) were treated as in (**A**). (**C**) HF were either left untreated or treated with mirin for one hour and then left untreated (mock) and HB-EGF was added for 5 min. Cells were lysed and subjected to Western blot analyses using the indicated antibodies as above. pEGFR signal intensities were quantified and normalized as in Fig. 1 (n = 5). Representative results of at least three independent experiments are shown.

To confirm the role of the Mre-11 and MRN complex in DNA damage-controlled EGFR activation, we assessed the effect of mirin^25^ on ligand-induced EGFR activation in normal fibroblasts. Mirin is an inhibitor of Mre-11 exonuclease activity that prevents MRN-dependent activation of ATM^25,26^. Notably, mirin prevented HB-EGF-induced phosphorylation of the EGFR and ERK in bleomycin-treated (Fig. 2A). In contrast, treatment with the inhibitors blocking either ATM (Ku55933) or DNA-PK (Nu7026) had only minor effects on the specific activation induced by HB-EGF (Fig. S2A). Similar results were obtained in peroxide-treated cells (Fig. S2B,C). Moreover, immunofluorescence shows translocation of phosphorylated ERK to the nucleus upon HB-EGF treatment, which is significantly reduced when cells are treated with mirin (Fig. S2D).

To rule out off-target effects of mirin, we additionally used ATLD2 cells. As expected, in Mre-11^−/−^ ATLD2 cells mirin (that act by binding to the active site of the Mre-11) had no effect on HB-EGF-induced EGFR signaling (Fig. 2B, left panel). However, when these cells were reconstituted with wild-type Mre-11^27^, the effect of mirin on ligand-dependent EGFR signaling was recovered (Fig. 2B, right panel), suggesting that the inhibition is specific for Mre-11 and not merely inhibiting EGFR activation. Moreover, the effect of mirin on EGFR, AKT, and ERK phosphorylation was specific for DNA damaged cells as no inhibition of HB-EGF-induced EGFR-signaling was observed in mock-treated controls (Fig. 2C).

Taken together these data revealed a link between nuclear Mre-11 in cells suffering from DNA damage and ligand-induced activity of surface EGFR.

### Regulation of Mig-6 expression by DNA damage

**Figure 3:**
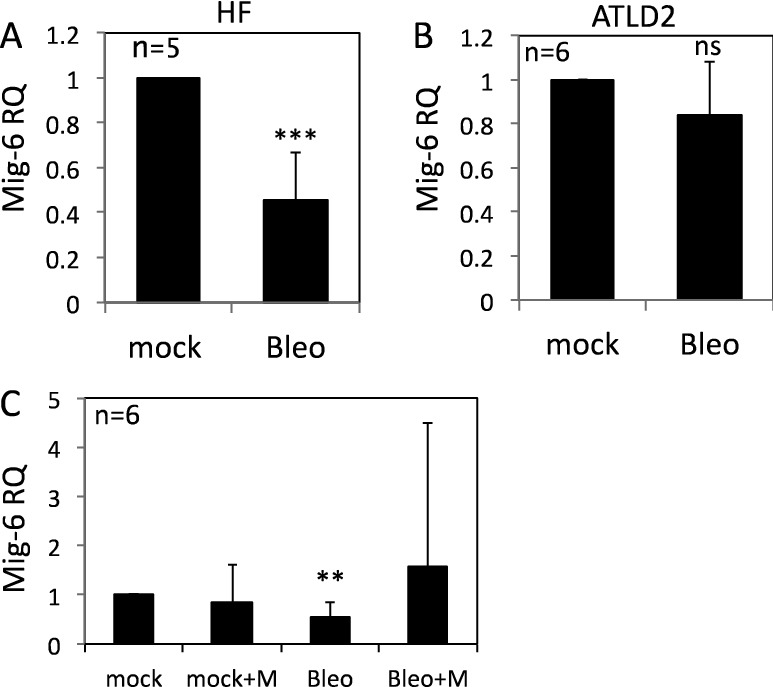
Regulation of Mig-6 expression by DNA damage. HF cells (**A**), ATLD2 cells (**B**), or HF cells that were pre-treated with mirin (100 µM) for an hour (**C**), were either left untreated or treated with bleomycin for 30 min and then RNA was extracted. Mig-6 mRNA expression level was determined by qRT-PCR and normalized to GAPDH. Graphs show an average (± STD) of the indicated number of independent experiments in each group. The expression of Mig-6 in control cells was set as 1. **p < 0.005; ***p < 0.0001; ns: non-significant.

Mig-6 is a cytoplasmic protein that functions as a feedback negative regulator of the EGF receptor-family^28^. Importantly, Mig-6 knockdown led to a significant increase in pEGFR (Y1173) as well as in pAKT and pERK in response to EGF treatment, whereas reconstitution of Mig-6 expression attenuated the activation of EGFR and the downstream signaling pathway^29,30^. Hence, we reasoned that Mig-6 might mediate the regulation of EGFR signaling by DNA damage. Consistent with this assumption, Mig-6 RNA expression was markedly reduced in fibroblasts following bleomycin treatment (Fig. 3A) but not in ATLD2 cells (Fig. 3B), which corresponded with the level of EGFR phosphorylation in these cells (Fig. 1). Moreover, Mig-6 downregulation was abrogated in mirin-treated cells. Although not statistically significant, Mig-6 was even elevated following bleomycin treatment (Fig. 3C) suggesting that upon DNA damage, Mre-11 may be responsible for the decrease in Mig-6 expression and consequently enhanced EGFR activation.

### DNA damage alters HB-EGF-induced gene expression profile

To test how DDR skews cellular response to EGFR activation, we assessed gene expression after treatment with HB-EGF following bleomycin mediated DNA damage in comparison to controls treated with HB-EGF alone. RNA extracted from cells treated with HB-EGF for 30 and 90 min were subjected to RNA sequencing (RNA-seq; Fig. S3A).

**Figure 4:**
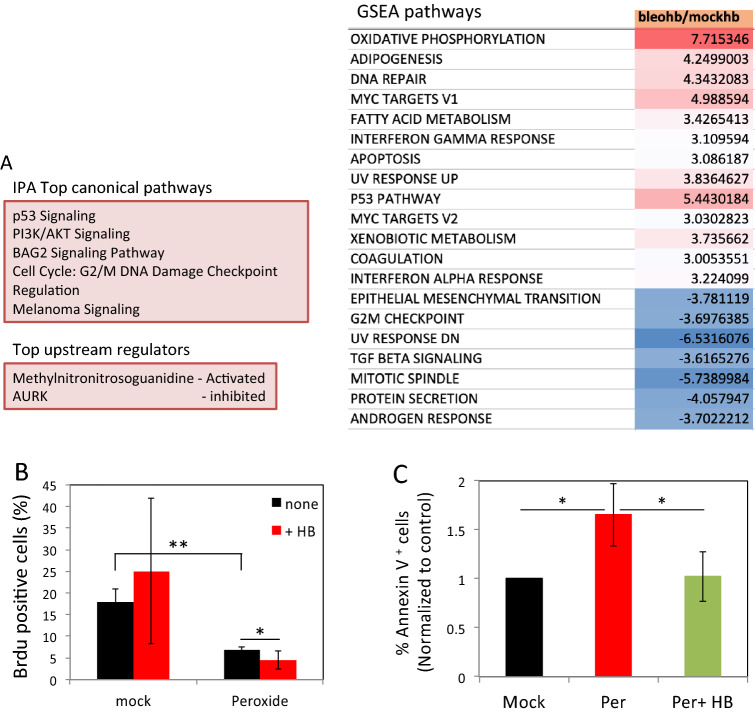
DNA damage alters gene expression profile and cellular response to HB-EGF triggering. (**A**) HF were treated as in Fig. 1A. After 30 and 90 min RNA was extracted and was subjected to RNAseq analysis. Differentially expressed genes altered with higher than twofold changes were selected. A list of IPA canonical pathways that are enriched in bleomycin treated cells stimulated with HB-EGF compared with those from HB-EGF stimulated mock cells (upper left panel). IPA upstream functional analysis was used to predict the top upstream regulators from differentially expressed genes (lower left panel). Gene set enrichment analysis (GSEA) pathways that are up-regulated (red) or down-regulated (blue) in response to HB-EGF treatment in bleomycin as compared with mock-treated cells (right panel). (**B**) Mock and peroxide (500 µM) treated cells were pulsed with BrdU for two hours immediately after treatment. After 24 h the cells were fixed and analyzed by flow cytometry. The percentage of BrdU positive cells is shown. Representative results of three independent experiments are shown. (**C**) Cells were treated as above and after 12 h were collected and stained with Annexin-V. The percentage of Annexin-V positive cells of three independent experiments was normalized to mock (set as 1). *p < 0.05; **p < 0.005; ns: non-significant.

Overall, RNA-Seq analysis identified more differentially expressed genes (DEG) following bleomycin treatment. A total of 7 and 45 DEG were identified between 30 and 90 min in mock and bleomycin treated cells, and 48 and 146 DEG were identified in response to HB-EGF treatment in the two groups, respectively (Fig. S3B). Significantly, only a minor overlap between genes differentially expressed in response to HB-EGF could be observed (Fig. S3C), suggesting that EGFR triggering induces expression of different gene sets in damaged cells as compared to mock treated controls. Indeed, Canonical Ingenuity Pathway Analysis (IPA) of DEG at 90 min and Gene set enrichment analysis (GSEA) revealed a clear difference in signaling pathways activated by HB-EGF in the context of DNA damage as compared to control cells (Figs. 4A and S3D). These analyses revealed that HB-EGF treatment in cells suffering from DNA damage reinforced the activation of DNA damage repair, UV response, G2/M DNA damage checkpoint, PI3K/AKT signaling and p53 pathways. On the other hand, cell cycle related pathways (such as mitotic spindle and G2 checkpoint) were reduced (Figs. 4A and S3D–F). Of note, the interferon-alpha response pathway induced by HB-EGF in bleomycin-treated cells was recently associated with p53 activation^31^.

To validate these RNAseq findings relating to cell cycle arrest, we analyzed 5-Bromo-2′-deoxyuridine (BrdU) incorporation into replicating DNA in cells suffering from DNA damage to examine the effect of HB-EGF on cell-cycle arrest. We specifically used peroxide as this treatment induces a high number of DNA breaks and causes complete block in cell-cycle^12^. In the first two hours, a decline in BrdU incorporation is observed in peroxide treated cells as compared to control cells. Consistent with the RNAseq analysis, this decline in BrdU incorporation is further intensified in the presence of HB-EGF (Fig. 4B).

After DNA damage the cell cycle is blocked to allow time for repair. However, depending on the extent of DNA damage, apoptosis can be induced (reviewed in^32^). Therefore, we tested the effect of EGFR triggering on DNA damage-induced cell death using Annexin-V staining. The percentage of Annexin-V positive cells increased significantly following peroxide treatment as compared to mock-treated cells, peaking at 12 h after treatment (Fig. S3G). Interestingly, HB-EGF rescued cells from peroxide-induced cell death (Figs. S3G and 4C).

Overall these results demonstrate that the cellular response to EGFR triggering is skewed in cells suffering from DNA damage and is characterized with augmented DNA damage response, p53 pathway, and a transient cell-cycle arrest. These changes are necessary for effective DNA repair and are consistent with improved DSB resolution in HB-EGF treated cells^12^.

## Discussion

Following DNA damage, cells activate the DDR cascade that largely relies on intra-nuclear protein networks, sensing DNA lesions and coordinating DNA repair. The Mre-11-RAD50-NBS1 (MRN) complex is one of the first sensors of DNA damage that plays a crucial role in orchestrating DNA damage response^8^. The DDR also communicates with the cell-cycle machinery and induces a transient p53-mediated cell-cycle arrest, which provides time for DNA repair^33^.

**Figure 5:**
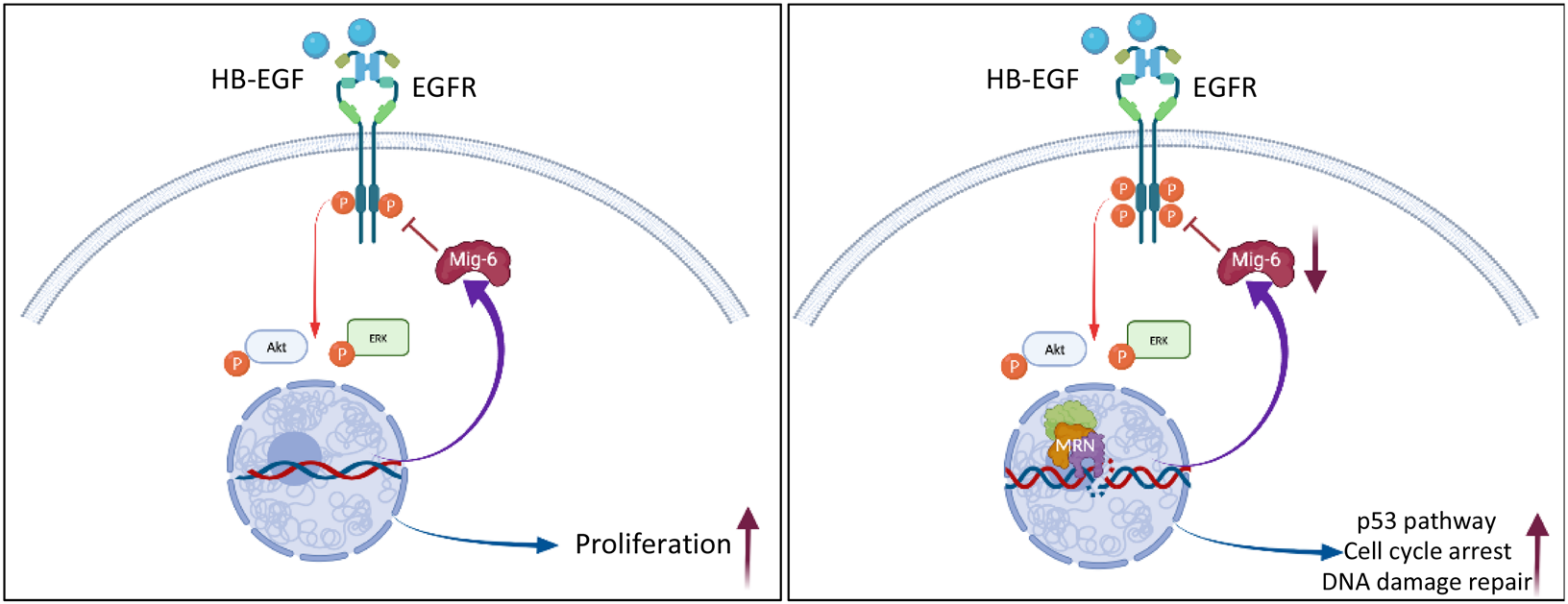
Model effects of DNA damage and repair on EGFR signaling and cellular response. (**A**) Under homeostatic conditions binding of ligand to EGFR induces canonical signals supporting cell proliferation and survival. (**B**) Upon induction of DNA damage, the MRN complex is assembled around sites of DSBs leading to a decrease in Mig-6 expression and amplifies ligand-induced EGFR signaling. In turn, amplified EGFR signaling enhances DDR-induced p53 pathway and cell cycle arrest and facilitates DSBs rejoining. Images created using BioRender.

In addition to its pro-proliferative activity, EGFR has been shown to enhance DDR signaling and to facilitate DNA damage repair^11,12^. However, the question arises as to how the two conflicting activities of cell-cycle progression driven by EGFR and the growth arrest necessary for effective DNA repair can be reconciled. It is reasonable to assume that stress conditions, such as DNA damage, within a cell may require a rapid and flexible strategy of diversifying the response to external stimuli. Our analysis demonstrates how such scenario can be generated, namely DDR modifies ligand-induced EGFR-ERK-AKT signaling pathway (Fig. 5).

Our study reveals that the DDR pathway biased ligand-induced EGFR-signaling network and gene expression profiles, which may provide an adaptive advantage for DNA-damaged cells. This novel pathway depends on intact MRN complexes affecting the expression of Mig-6 to coordinate DNA damage repair with response to extracellular signals. We suggest that this bidirectional crosstalk of nuclear (DDR) and extracellular signals (EGFR) is required to synchronize and coordinate cell-cycle arrest and DNA repair.

Specifically, our data demonstrate that ligand-induced EGFR phosphorylation and two well-characterized downstream signaling pathways, ERK and AKT, are enhanced in cells suffering from DNA damage. Importantly, DNA damage modulates the biological responses to ligand-induced EGFR signaling. In contrast to the pro-proliferative activity of EGFR signaling, triggering this pathway in cells suffering from DNA damage augments the canonical DNA damage-induced p53 pathway and reinforces transient cell growth arrest and efficient DNA damage repair. Notably, a recent study has demonstrated that upon DNA damage ATM stabilizes the transforming growth factor β (TGFβ) receptor, which in turn execute its anti-proliferative activity and thus contribute to cell cycle arrest^34^. However, our study presents an even more complex scenario, whereby DNA damage modifies EGFR signaling and changes the cellular response to EGFR triggering, strengthen p53 and transient cell cycle arrest. Interestingly, several studies have demonstrated that in subsets of cancer cells, and specifically breast cancer cells, EGFR function changes from proliferative in primary tumors to growth-inhibitory and apoptotic in metastatic tumors^35–39^. Moreover, a biased downstream ligand-induced EGFR signaling and the activation of STAT1 were shown to be responsible for enhanced apoptosis and growth inhibition in the metastatic cells^40^.

The diversity of signaling networks activated by ligand-dependent activation of EGFR prevents the logical elucidation of how the biochemical signal links to the appropriate biological responses. However, it would be reasonable to argue that the duration and strength of signals that are tightly regulated by the action of numerous negative regulatory mechanisms, such as Mig-6, is translated into different gene expression profiles that could convert cellular function. A previous study suggested that unbalanced expression levels of Mig-6 with relation to EGFR lead to persistent activation of EGFR that initiates a pro-apoptotic cascade^39^.

Upon ligand binding, Mig-6 associates with the intracellular domain of EGFR and inhibits EGFR signaling via two mechanisms that involve receptor degradation and kinase suppression^28,41–43^. Thus, reduced Mig-6 is expected to confer higher sensitivity to EGFR triggering. Our data demonstrate that DNA damage reduces Mig-6 expression and heightens EGFR phosphorylation and activation. Importantly, these DNA damage-dependent modifications in ligand-induced EGFR activation did not involve any changes in EGFR surface expression, as would be expected from a mechanism involving receptor degradation.

The regulation of EGFR activation by DNA damage was dependent on the expression of Mre-11 and presumably on the assembly of an active MRN complex upon DNA damage. Mre-11 is an important component of the Mre-11-RAD50-NBS1 (MRN) complex that plays a crucial role in DDR, as it is one of the first sensors and responders to DNA damage. Through interacting with other players at the double-strand break, the MRN complex initiates and amplifies DDR signaling and orchestrates DNA damage repair pathways^44,45^. In addition, to produce the appropriate biological responses DNA damage can positively regulate transcription initiation of specific transcriptional programs^46–48^. Interestingly, we observed a change in Mig-6 mRNA levels shortly after the induction of DNA damage, thus coupling DDR function to signaling through cell surface receptors. A previous study demonstrated that ligand-induced activation of the EGFR is defective in ataxia-telangiectasia (A-T) cells^49^, suggesting a role for ATM in EGFR activation. Similarly, ATM has been shown to play a role in the insulin receptor-signaling pathway that controls translation initiation^50^. However, in contrast to our findings, these cytoplasmic functions of ATM in regulating surface receptor signaling are taking place in control cells and are independent of its role in DNA damage.

In summary, we described an ‘inside-out’ signaling, originating from the DNA lesion to modify signal transduction triggered by EGFR activation. This allows the cells to survive and to perform efficient DNA repair. Thus, this study provides a general framework in which cells re-wire cellular networks in response to external cues mediated by surface receptors according to their cellular condition.

## Materials and methods

### Cells

Primary normal human dermal fibroblasts (HF) were provided by the Department of Surgery, Hadassah Hebrew-University Medical Center, Jerusalem, Israel. ATLD2 fibroblasts and ATLD cells complemented with wild-type Mre-11 (a generous gift from Dr. Yosei Shiloh, Tel Aviv University, Israel). Cells were maintained (not more than 10 passages) in a high-glucose DMEM medium (Life Technologies, Grand Island, NY). All media were supplemented with 10% heat-inactivated fetal calf serum, 1% sodium pyruvate, and 1% penicillin/streptomycin (Biological Industries, Beit-Haemek, Israel) at 37 °C and 5% CO_2_.

### Experimental protocol

The in-vitro experimental protocol is adapted from our previous work^12^. For DNA damage induction cells were treated with either bleomycin (15 µg/ml) or hydrogen peroxide (EMD Millipore Corp. Billerica, MA; 500 µM H_2_O_2_ in PBS) or were mock-treated for 30 min, and were then washed with fresh media. Only at that stage, HB-EGF (PeproTech, Rocky Hill, NJ; 10 ng/ml) was added, and cells were incubated for the indicated time points.

### Cell lysis and immunoblotting

Cells were lysed with X3 LDS-PAGE sample buffer (GeneScript, Piscataway, NJ). The lysates were boiled for 30 min and then H_2_O (1:3 v/v) was added and samples were boiled for an additional 5 min. Samples were kept at − 20 °C until use. Lysates were separated by electrophoresis on 4–20% gradient SurePAGE Bis–Tris gels (GeneScript) and Tris-MOPS-SDS running buffer (Gene Script) and then transferred to PVDF membranes using Trans-Blot Turbo Transfer Pack (Bio-Rad, Hercules, CA). The blots were probed with anti-phosphorylated EGFR (Ty1173; R&D systems), anti-phosphorylated ERK, or anti-phosphorylated AKT (Cell Signaling, Danvers, MA) followed by anti-Rabbit Envision^+^ System-HRP Labeled Polymer (Agilent Dako, Santa Clara, CA) and processed with Clarity™ Western ECL substrate (Bio-Rad). Signal was detected using the ChemiDoc™ MP imaging system (Bio-Rad). Following stripping, the membranes were re-probed with anti-Actin mAb (MP Biomedicals, LLC, Illkirch, France). Quantitation of bands was performed using Image-Lab software (Bio-Rad).

In all figures presenting Western blots: the images in each figure are from the same experiment and treated under the same conditions (blotting, antibody hybridization exposure time, etc.). No contrast or other visual manipulation was preformed. Where ever images contain blots that were cropped from different areas of the membrane it is clearly defined by dividing lines.

A Human EGFR Phosphorylation Antibody Array (Abcam, Cambridge, MA) was used to detect the relative levels of 17 different phosphorylated Human EGF Receptors in cell and tissue lysates. Cell lysates were prepared and membrane handling and probing were preformed according to manufacturer instructions. Signal detection and quantitation were performed using Image-Lab software (Bio-Rad). Signal intensity was normalized according to manufacturer instructions.

### Flow cytometry

HF were trypsinized and immuno-stained using Alexa-488 conjugated anti-EGFR human recombinant mAb (research-grade Cetuximab, R&D) for 30 min on ice. Cells were washed twice in PBS, and the immune-stained cells were analyzed by a CytoFLEX Flow cytometer (Beckman Coulter, Indianapolis, USA) using the CytExpert software.

### RNA extraction

Total RNA was isolated from cells using Trizol reagent (Life Technologies, Paisley, UK) and purified using MaXtract High Density (QIAGEN, Redwood City, CA). RNA yield and quantity were determined using a Nanodrop spectrophotometer ND-1000 (Thermo Scientific, Wilmington, DE). The RNA quality was tested using an ND-1000 V3.7.1, according to the manufacturer’s instructions, and assigned an RNA integrity number (RIN). Only samples with an RNA integrity number (RIN) of 8 or greater were employed as was previously described^51^.

### Mig-6 mRNA expression analysis

As was previously described^52^ total RNA was reverse transcribed to cDNA using oligo(dT) primers and M-MLV reverse transcriptase (Thermo Scientific). PerfeCTa SYBR Green FastMix ROX (Quanta Biosciences) was used for real-time PCR according to the manufacturer’s protocol and all the samples were run in triplicate on CFX384 Touch Real-Time system c1000 thermal cycler (Bio-Rad, Hercules, CA). Cycling conditions were 95 °C for 20 s, followed by 40 cycles of 95 °C for 1 s, and 60 °C for 20 s, 65 °C for 5 s. Gene expression levels were normalized to the GAPDH gene. Primers used for qPCR analysis are: Mig-6 sense-GGAGCAGTCGCAGTGAGTTT; anti-sense-GGAAGCATGCCCAAGTGGTA; GAPDH sense-AGCCTCAAGATCATCAGCAATG; anti-sense-CACGATACCAAAGTTGTCATGGAT.

### RNAseq analysis

Each sample is represented by three replicates of an equal amount of total RNA from three samples that were pooled before library construction. RNAseq libraries were constructed in the Technion Genome Center using Illumina TruSeq RNA Library Preparation Kit v2 according to Illumina protocol and sequenced with the Illumina HiSeq 2500 System to an average depth of around 20 million reads per sample. The bioinformatics analyses were performed in the Technion Genome Center. Raw reads were first pre-processed using trim_galore (cutadapt version 1.10) to remove adapter and low-quality sequence. The processed fastq files were mapped to the human transcriptome and genome using TopHat (v2.1.0) using Bowtie2 [version 2.2.6]. The genome version was GRCh38, with annotations from Ensembl release 95. Transcript counts were quantified with HTSeq [version 0.11.2] and differential gene expression was analyzed using the Bioconductor package DESeq2 [version 1.24.0]. Genes with an adjusted p-value below 0.01 were considered significantly differentially expressed. Analysis for enriched pathways, upstream regulators and networks were performed using QIAGEN’s Ingenuity^®^ Pathway Analysis (IPA^®^, QIAGEN, http://www.qiagen.com/ingenuity).

Gene set enrichment analysis (GSEA^53^) was performed the I-CORE Bioinformatics Unit of the Hebrew University of Jerusalem and Hadassah Medical Center for Bioinformatics data analysis using whole differential expression data (cut-off independent) to determine whether a priori-defined sets of genes show statistically significant, concordant differences between two biological states. The hallmark gene set collection from the molecular signatures database (MsigDB version 7.1), was used. The GEO accession number for RNAseq dataset reported in this paper is GSE183269.

### Apoptosis assay

Quantification of apoptotic cells was performed using TACS Annexin V-FITC Apoptosis Detection Kit (R&D Systems) according to manufacturer instructions.

### BrdU incorporation assay

To assess the effect of DNA damage and HB-EGF on cell proliferation rate, the BrdU incorporation assay. HF were seeded and exposed to either peroxide or bleomycin and then HB-EGF was added and cells were labeled with 10 μM of BrdU (Sigma Aldrich; MA, USA) solution for 2 h, the labeling medium was then removed and cells were incubated for an additional 24 h. Control samples were grown without BrdU supplement. At the end of treatment cells were detached, washed and fixed using cold absolute ethanol and stored overnight at − 20 °C. For DNA denaturation, fixed cells were incubated for 30 min in RT with denaturation buffer (2 N HCl in 0.5% triton) that was neutralized using neutralization buffer (0.1 M Na_2_B_4_O_7_, pH8.5) and then washed with PBS. For BrdU detection, cells were incubated with 15 μg/mL anti-BrdU mAb (clone BRD.3; NeoMarkers; CA, USA) Overnight at 4 °C and then washed, and incubated with 5 μg/mL Alexa fluor 488-conjugated F (ab′) 2 fragment goat anti-mouse IgG (Invitrogen; CA, USA) for 30 min at 4 °C.

### Statistical analysis

All data were subjected to statistical analysis as described^52^, using the Excel software package (Microsoft) or GraphPad Prism6 (GraphPad Software Inc., La Jolla, CA, USA). A two-tailed Student’s t-test was used to determine the difference between the groups. Data are given as mean ± SD and are shown as error bars for all experiments. Differences were considered significant at P < 0.05.

## Supplementary Information

Supplementary Figures.

Supplementary Information 2.

## References

[1] Dinkelmann M (2009). Multiple functions of MRN in end-joining pathways during isotype class switching. Nat. Struct. Mol. Biol..

[2] Rass E (2009). Role of Mre11 in chromosomal nonhomologous end joining in mammalian cells. Nat. Struct. Mol. Biol..

[3] Taylor EM (2010). The Mre11/Rad50/Nbs1 complex functions in resection-based DNA end joining in *Xenopus laevis*. Nucleic Acids Res..

[4] Guo X (2008). Development of a real-time PCR method for Firmicutes and Bacteroidetes in faeces and its application to quantify intestinal population of obese and lean pigs. Lett. Appl. Microbiol..

[5] Reginato G, Cejka P (2020). The MRE11 complex: A versatile toolkit for the repair of broken DNA. DNA Repair.

[6] Falck J, Coates J, Jackson SP (2005). Conserved modes of recruitment of ATM, ATR and DNA-PKcs to sites of DNA damage. Nature.

[7] Stracker TH, Petrini JH (2011). The MRE11 complex: Starting from the ends. Nat. Rev. Mol. Cell Biol..

[8] Williams RS, Williams JS, Tainer JA (2007). Mre11-Rad50-Nbs1 is a keystone complex connecting DNA repair machinery, double-strand break signaling, and the chromatin template. Biochem. Cell Biol..

[9] Wyman C, Kanaar R (2006). DNA double-strand break repair: All's well that ends well. Annu. Rev. Genet..

[10] Fernandez-Capetillo O, Lee A, Nussenzweig M, Nussenzweig A (2004). H2AX: The histone guardian of the genome. DNA Repair.

[11] Meyn RE, Munshi A, Haymach JV, Milas L, Ang KK (2009). Receptor signaling as a regulatory mechanism of DNA repair. Radiother. Oncol..

[12] Geiger-Maor A (2015). Macrophages regulate the systemic response to DNA damage by a cell non-autonomous mechanism. Cancer Res..

[13] Schlessinger J (2014). Receptor tyrosine kinases: Legacy of the first two decades. Cold Spring Harb. Perspect. Biol..

[14] Yarden Y, Pines G (2012). The ERBB network: At last, cancer therapy meets systems biology. Nat. Rev. Cancer.

[15] Sabbah DA, Hajjo R, Sweidan K (2020). Review on epidermal growth factor receptor (EGFR) structure, signaling pathways, interactions, and recent updates of EGFR inhibitors. Curr. Top. Med. Chem..

[16] Kriegs M (2010). The epidermal growth factor receptor modulates DNA double-strand break repair by regulating non-homologous end-joining. DNA Repair.

[17] Burdak-Rothkamm S (2005). Radiosensitivity of tumor cell lines after pretreatment with the EGFR tyrosine kinase inhibitor ZD1839 (Iressa). Strahlenther Onkol..

[18] Mukherjee B (2009). EGFRvIII and DNA double-strand break repair: A molecular mechanism for radioresistance in glioblastoma. Cancer Res..

[19] Steelman LS (2020). Therapeutic resistance in breast cancer cells can result from deregulated EGFR signaling. Adv. Biol. Regul..

[20] Jost M, Kari C, Rodeck U (2000). The EGF receptor—An essential regulator of multiple epidermal functions. Eur. J. Dermatol..

[21] Chen J, Ghorai MK, Kenney G, Stubbe J (2008). Mechanistic studies on bleomycin-mediated DNA damage: Multiple binding modes can result in double-stranded DNA cleavage. Nucleic Acids Res..

[22] Povirk LF (1996). DNA damage and mutagenesis by radiomimetic DNA-cleaving agents: Bleomycin, neocarzinostatin and other enediynes. Mutat. Res..

[23] Lemmon MA, Schlessinger J, Ferguson KM (2014). The EGFR family: Not so prototypical receptor tyrosine kinases. Cold Spring Harb. Perspect. Biol..

[24] Stewart GS (1999). The DNA double-strand break repair gene hMRE11 is mutated in individuals with an ataxia-telangiectasia-like disorder. Cell.

[25] Dupre A (2008). A forward chemical genetic screen reveals an inhibitor of the Mre11-Rad50-Nbs1 complex. Nat. Chem. Biol..

[26] Shibata A (2014). DNA double-strand break repair pathway choice is directed by distinct MRE11 nuclease activities. Mol. Cell.

[27] Uziel T (2003). Requirement of the MRN complex for ATM activation by DNA damage. EMBO J..

[28] Hackel PO, Gishizky M, Ullrich A (2001). Mig-6 is a negative regulator of the epidermal growth factor receptor signal. Biol. Chem..

[29] Ying H (2010). Mig-6 controls EGFR trafficking and suppresses gliomagenesis. Proc. Natl. Acad. Sci. USA..

[30] Li Z (2012). Downregulation of Mig-6 in nonsmall-cell lung cancer is associated with EGFR signaling. Mol. Carcinog..

[31] Zhang W (2020). The mitochondrial protein MAVS stabilizes p53 to suppress tumorigenesis. Cell Rep..

[32] Chen J (2016). The cell-cycle arrest and apoptotic functions of p53 in tumor initiation and progression. Cold Spring Harb. Perspect. Med..

[33] Hartwell LH, Weinert TA (1989). Checkpoints: Controls that ensure the order of cell cycle events. Science.

[34] Li Y (2019). DNA damage activates TGF-beta signaling via ATM-c-Cbl-mediated stabilization of the type II receptor TbetaRII. Cell Rep..

[35] Ali R, Wendt MK (2017). The paradoxical functions of EGFR during breast cancer progression. Signal Transduct. Target Ther..

[36] Choi J (2010). Epidermal growth factor induces cell death in the absence of overexpressed epidermal growth factor receptor and ErbB2 in various human cancer cell lines. Cancer Investig..

[37] Jackson NM, Ceresa BP (2016). Protein kinase G facilitates EGFR-mediated cell death in MDA-MB-468 cells. Exp. Cell Res..

[38] Lim YJ, Jeon SR, Koh JM, Wu HG (2015). Tumor growth suppression and enhanced radioresponse by an exogenous epidermal growth factor in mouse xenograft models with A431 cells. Cancer Res. Treat..

[39] Wendt MK (2015). The antitumorigenic function of EGFR in metastatic breast cancer is regulated by expression of Mig6. Neoplasia.

[40] Ali R, Brown W, Purdy SC, Davisson VJ, Wendt MK (2018). Biased signaling downstream of epidermal growth factor receptor regulates proliferative versus apoptotic response to ligand. Cell Death Dis..

[41] Anastasi S, Lamberti D, Alema S, Segatto O (2016). Regulation of the ErbB network by the MIG6 feedback loop in physiology, tumor suppression and responses to oncogene-targeted therapeutics. Semin. Cell Dev. Biol..

[42] Frosi Y (2010). A two-tiered mechanism of EGFR inhibition by RALT/MIG6 via kinase suppression and receptor degradation. J. Cell Biol..

[43] Walsh AM, Lazzara MJ (2013). Regulation of EGFR trafficking and cell signaling by Sprouty2 and MIG6 in lung cancer cells. J. Cell Sci..

[44] Bian L, Meng Y, Zhang M, Li D (2019). MRE11-RAD50-NBS1 complex alterations and DNA damage response: Implications for cancer treatment. Mol. Cancer.

[45] Syed A, Tainer JA (2018). The MRE11-RAD50-NBS1 complex conducts the orchestration of damage signaling and outcomes to stress in DNA replication and repair. Annu. Rev. Biochem..

[46] Elkon R (2005). Dissection of a DNA-damage-induced transcriptional network using a combination of microarrays, RNA interference and computational promoter analysis. Genome Biol..

[47] Silva E, Ideker T (2019). Transcriptional responses to DNA damage. DNA Repair.

[48] Vitelli V (2017). Recent advancements in DNA damage-transcription crosstalk and high-resolution mapping of DNA breaks. Annu. Rev. Genomics Hum. Genet..

[49] Keating KE, Gueven N, Watters D, Rodemann HP, Lavin MF (2001). Transcriptional downregulation of ATM by EGF is defective in ataxia-telangiectasia cells expressing mutant protein. Oncogene.

[50] Yang DQ, Kastan MB (2000). Participation of ATM in insulin signalling through phosphorylation of eIF-4E-binding protein 1. Nat. Cell Biol..

[51] Guedj A, Geiger-Maor A, Galun E, Amsalem H, Rachmilewitz J (2016). Early age decline in DNA repair capacity in the liver: In depth profile of differential gene expression. Aging.

[52] Guedj A (2020). Gut microbiota shape 'inflamm-ageing' cytokines and account for age-dependent decline in DNA damage repair. Gut.

[53] Subramanian A (2005). Gene set enrichment analysis: A knowledge-based approach for interpreting genome-wide expression profiles. Proc. Natl. Acad. Sci. USA.

