# Supplementary material for: DNA damage alters EGFR signaling and reprograms cellular response via Mre-11
Source: Sci Rep. 2022 Apr 6;12:5760. doi: 10.1038/s41598-022-09779-5 (PMC8986772; doi:10.1038/s41598-022-09779-5)
Supplement: Supplementary file 1 — Supplementary Figures. [file 41598_2022_9779_MOESM1_ESM.pdf]

SUPPLEMENTARY FIGURES

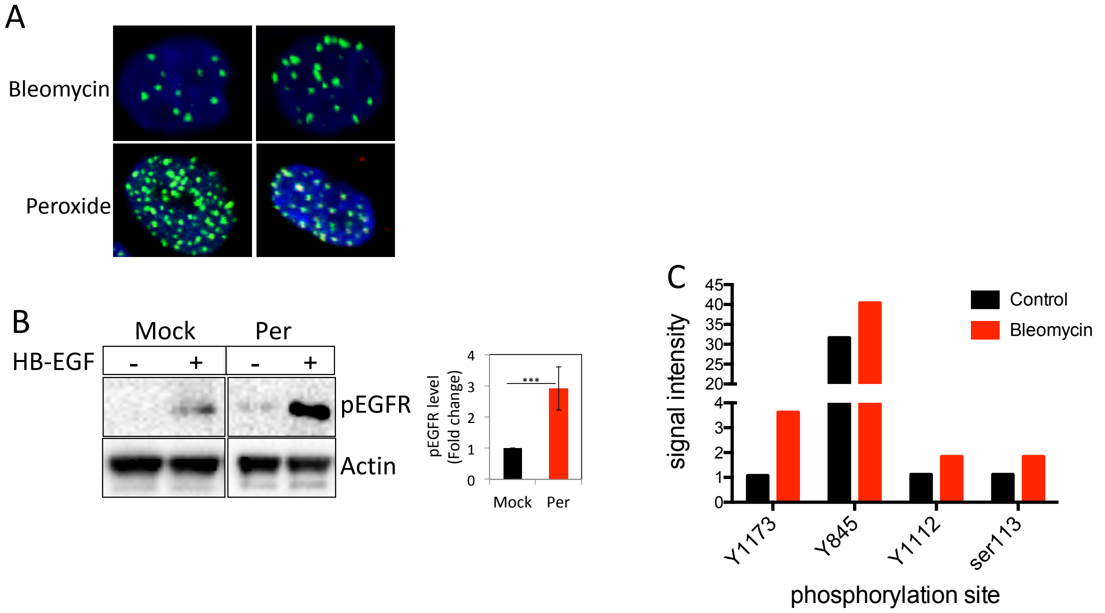

Supplementary Figure S1: DNA damage enhances ligand-induced EGFR phosphorylation. A. Representative confocal immunofluorescence images showing γH2AX foci (green) with DAPI counterstain (blue) in HF treated with either bleomycin (15 μg/ml) or peroxide (500 μM H_2O_2). Normal human dermal fibroblasts (HF) were fixed 30 min after treatment and were immunostained using anti-γH2AX mAb as previously described (11). **B.** HF were either left untreated or treated with peroxide (500μM) for 30 min, followed by extensive washing and subsequent exposure to HB-EGF (10 ng/ml) for 5 min. Western blot analyses were performed on cell extracts using antibodies to phosphorylated EGFR (Y1173). Anti-β-actin immunoblotting revealed relative amounts of protein in each lane. pEGFR signal intensities were quantified and normalized to actin signal (loading control). The level of EGFR phosphorylation in response to HB-EGF treatment in peroxide treated cells as compared to that of mock-treated cells (set as 1) are shown (n=5). Representative results of five independent experiments are shown. *** $p<0.001$ **C**. HF were treated with bleomycin followed by 5min treatment with HB-EGF as above. Cells were then lysed and lysates were analyzed and quantified using the Human EGFR Phosphorylation Antibody Array according to manufacturer instructions.

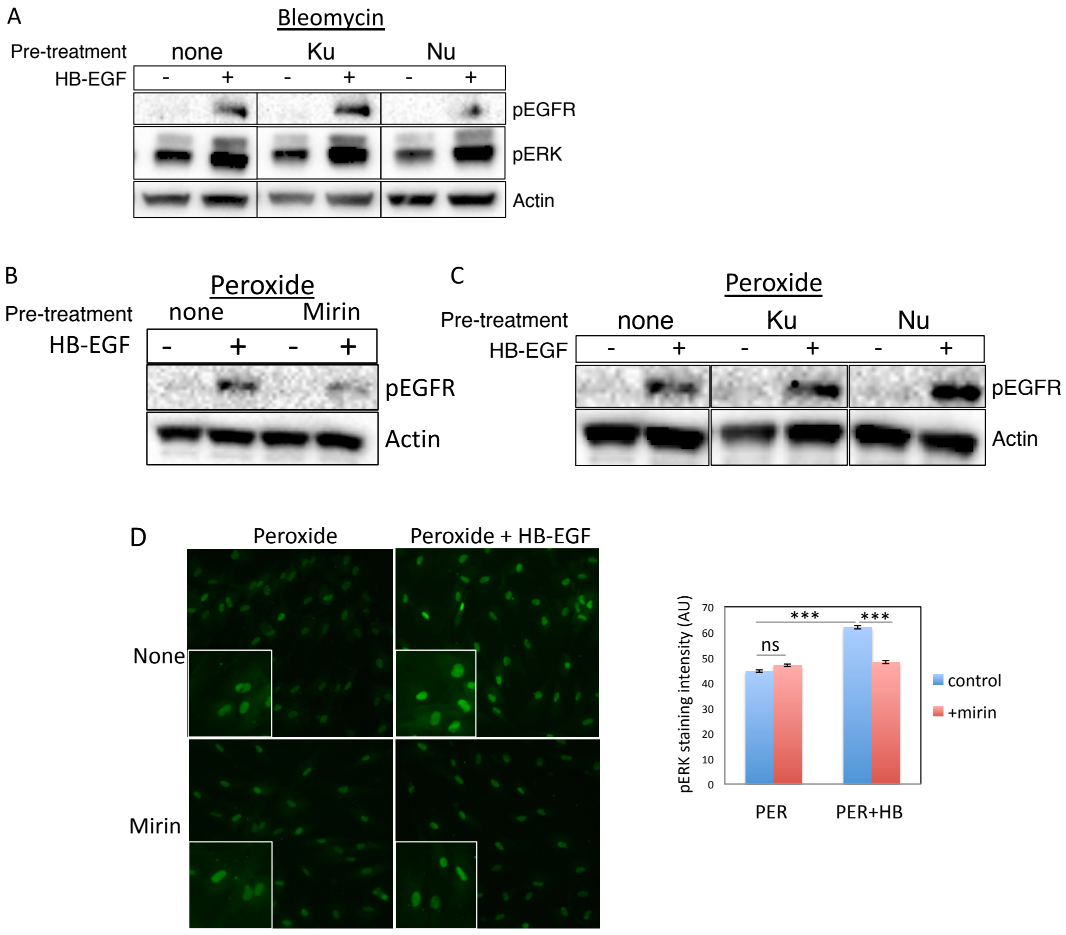

Supplementary Figure S2: **Pharmacological inhibition of mre-11 interferes with ligand-dependent EGFR signaling in cells suffering from DNA damage**

A. HF were either left untreated or treated with either Ku5593 (10µM) or Nu7026 (10µM) for one hour and then were treated with bleomycin for an additional 30 min followed by HB-EGF treatment for 5 min. **B.** Cells were treated with mirin as above and then treated with peroxide (500µM) for 30 min and following washing, cells were incubated with and without HB-EGF for 5 min. Cells were lysed and subjected to Western blot analyses using the indicated antibodies as above. **C.** HF were either left untreated or treated with either Ku5593 (10µM) or Nu7026 (10µM) for one hour and then were treated with peroxide for an additional 30 min followed by HB-EGF treatment for 5 min. pEGFR signal intensities were quantified and normalized to actin signal (loading control) and are depicted as fold change of EGFR phosphorylation following stimulation with HB-EGF. Representative results of three independent

experiments are shown. **D.** HF were treated as in C and then fixed and immunostained with anti-pERK mAbs. The graph on the right depicts pERK signal intensities. *** $p<0.0001$; ns: non-significant

A Expose cells to Bleomycin → Wash → Add medium without or with HB-EGF → Incubate for 30/90 min and extract RNA

B

Comparison	All_Zero	Low_Counts	Tested	Significant_Up	Significant_Down
mock30_vs_mock90	20,362	21,769	16,545	0	7
Bleo30_vs_Bleo90	20,362	21,666	16,648	3	42
mock_HB 30_vs_mock_HB90	20,362	21,773	16,541	15	33
Bleo_HB30_vs_Bleo_HB90	20,362	21,918	16,396	21	125

90 min

Comparison	All_Zero	Low_Counts	Tested	Significant_Up	Significant_Down
Bleo_HB_vs_Bleo	24,042	23,937	10,697	17	1
mock_HB_vs_mock	24,042	23,937	10,697	13	0
Bleo _vs_ mock	24,042	17,918	16,716	2	0
Bleo _HB_vs mock _HB	24,042	17,962	16,672	11	11

C

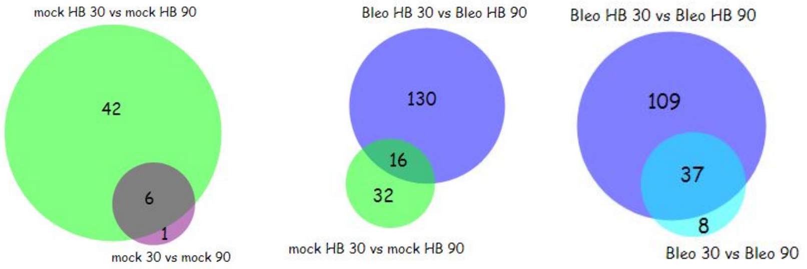

D

IPA Top canonical pathways

Cholecystokinin/Gastrin-mediated Signaling
ILK Signaling
IL-8 Signaling
MIF Regulation of Innate Immunity
Colorectal Cancer Metastasis Signaling

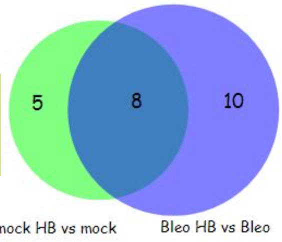

D-myo-inositol (1,4,5,6)-Tetrakisphosphate Biosynthesis
D-myo-inositol (3,4,5,6)-tetrakisphosphate Biosynthesis
Protein Kinase A Signaling
3-phosphoinositide Degradation
D-myo-inositol-5-phosphate Metabolism

GSEA pathways	mockhb/mock	bleohb/bleo
UV RESPONSE UP	0	3.8175259
P53 PATHWAY	0	4.57142
XENOBIOTIC METABOLISM	0	3.404037
COAGULATION	0	3.2013557
INTERFERON ALPHA RESPONSE	0	3.8921697
MITOTIC SPINDLE	0	-4.186181
HYPOXIA	3.2701132	0
IL2 STAT5 SIGNALING	3.3689992	0
ALLOGRAFT REJECTION	3.0312912	0

E

Bleo vs Mock

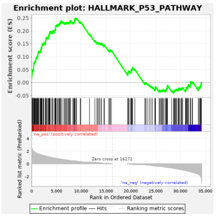

FDR: 0.0
NES: 4.1589584

Bleo HB vs Bleo

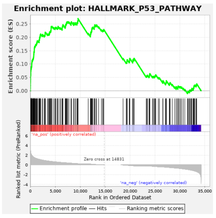

FDR: 0.0
NES: 4.57142

F

Bleo vs Mock

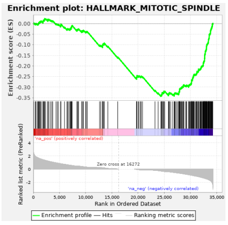

FDR: 0.0
NES: -5.637597

Bleo HB vs Bleo

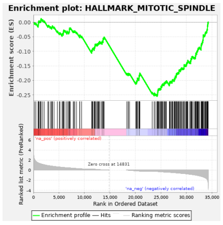

FDR: 0.0
NES: -4.186181

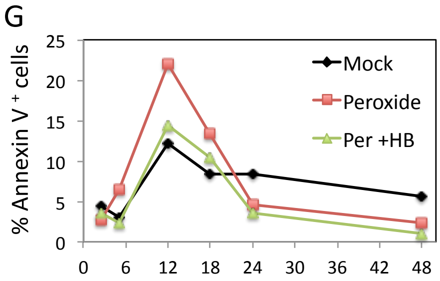

Supplementary Figure S3: DNA damage alters gene expression profile and cellular response to HB-EGF triggering

A. Timeline of the experimental procedure. **B.** Tables summarizing the numbers of differentially expressed genes between the different experimental groups and times. **C.** Venn diagrams comparing the number of genes specifically changing in response to HB-EGF in mock and bleomycin treated cells, demonstrating a marked difference in the genes involved in the response to HB-EGF in cells suffering from DNA damage as compared to control cells. **D.** A list of IPA canonical pathways that are enriched in bleomycin treated cells stimulated with HB-EGF (upper right panel) and those of HB-EGF stimulated mock cells (upper left panel). Gene set enrichment analysis (GSEA) pathways that are up-regulated (red) or down-regulated (blue) in response to HB-EGF treatment in bleomycin and mock-treated cells (lower panel). GSEA enrichment score curves of p53 pathway (**E**) and mitotic spindle (**F**) demonstrating that HB-EGF reinforces bleomycin-induced p53 activation and reduces cell cycle progression. **G.** Cells were either mock or bleocmycin treated and then HB-EGF was added. At the indicated time points, cells were collected and stained with Annexin-V. The percentage of Annexin-V positive cells is shown. One representative of three independent experiments is shown.
