# Supplementary material for: DNA damage alters EGFR signaling and reprograms cellular response via Mre-11
Source: Sci Rep. 2022 Apr 6;12:5760. doi: 10.1038/s41598-022-09779-5 (PMC8986772; doi:10.1038/s41598-022-09779-5)

Figure 1A pEGFR

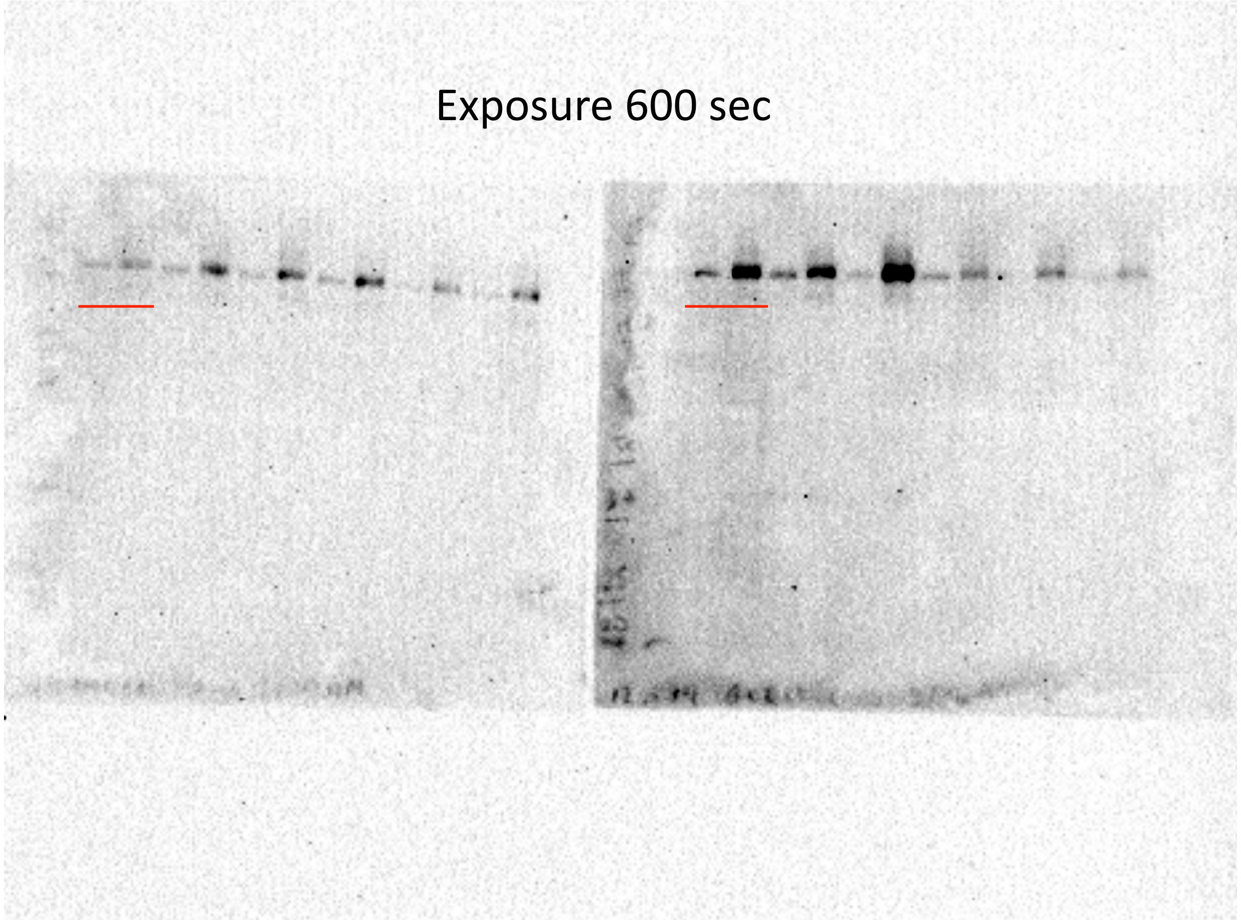

Exposure 120 sec

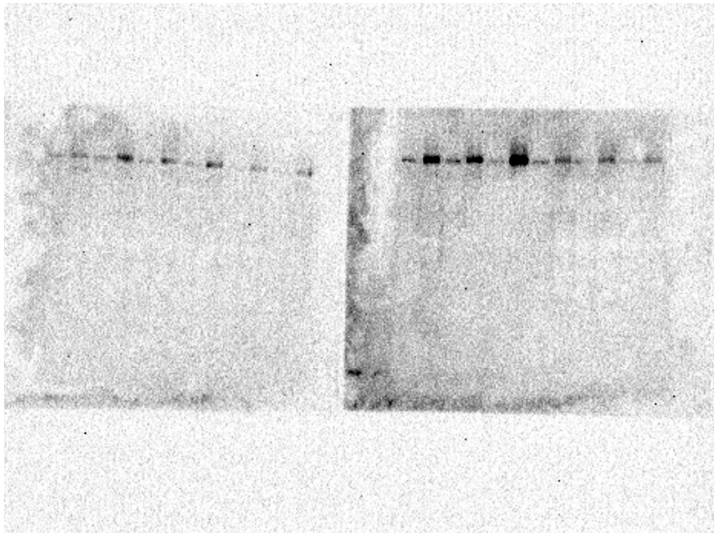

Figure 1A pAKT

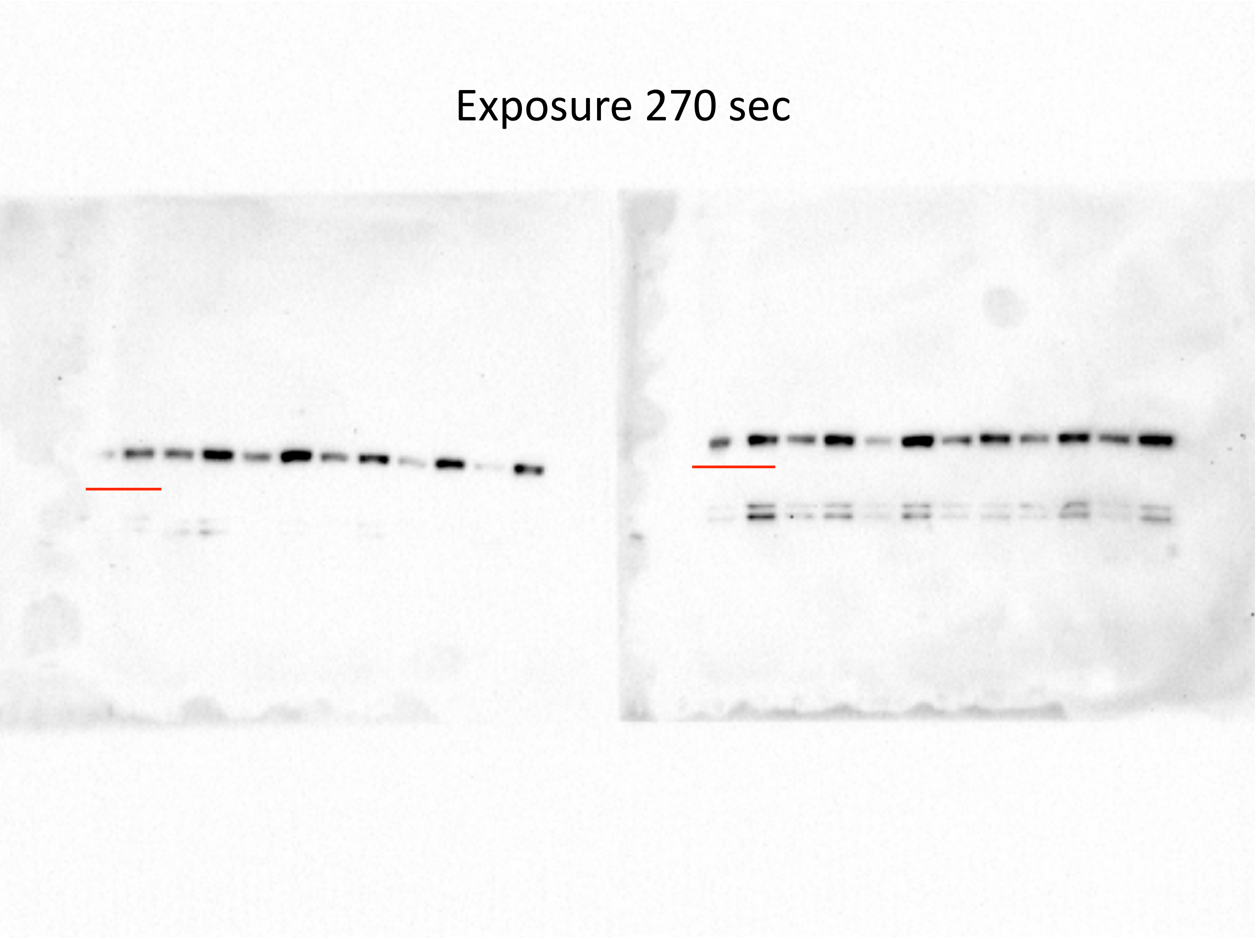

Exposure 30 sec

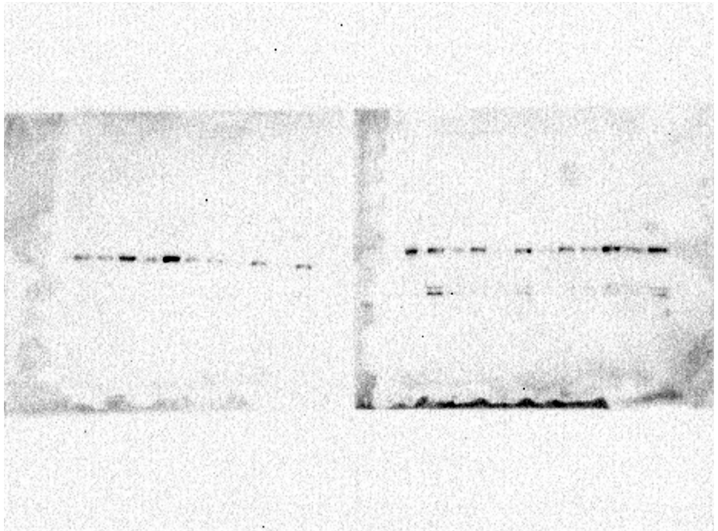

Figure 1A pERK

Exposure 9 sec

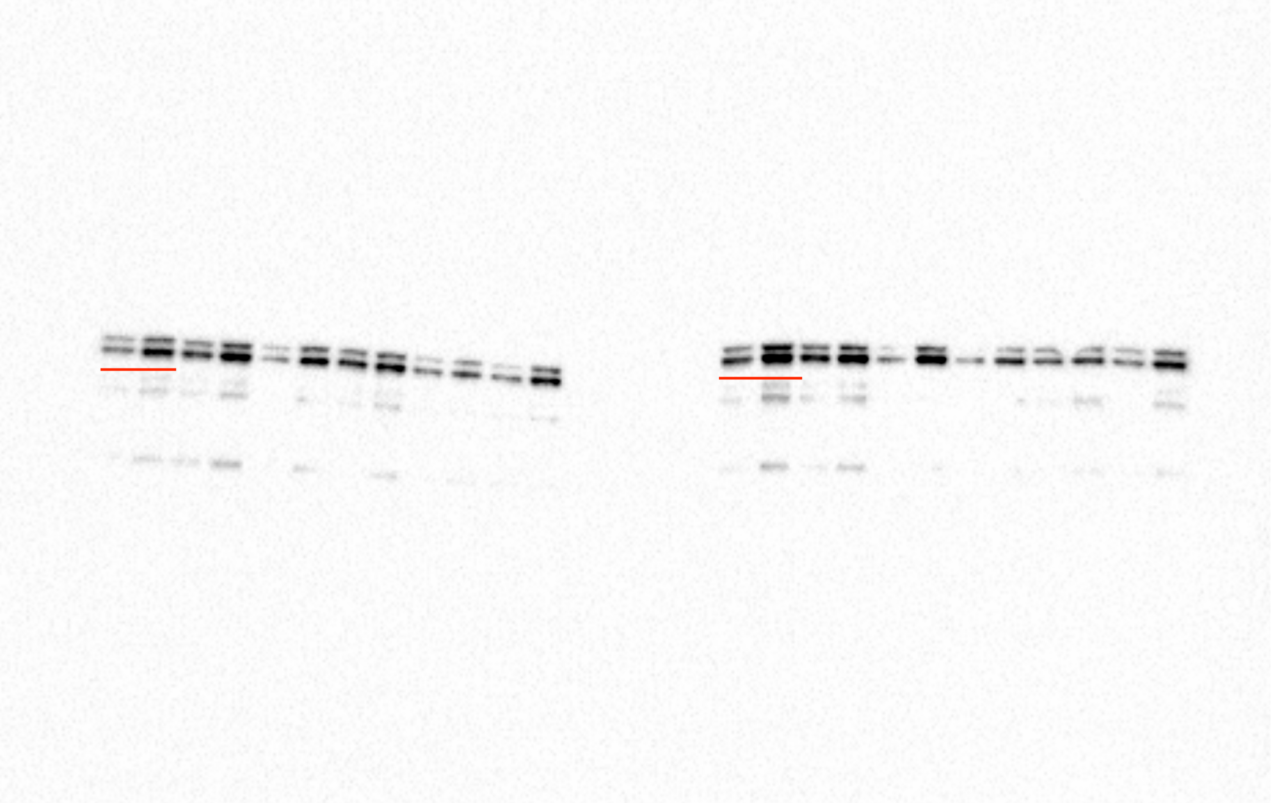

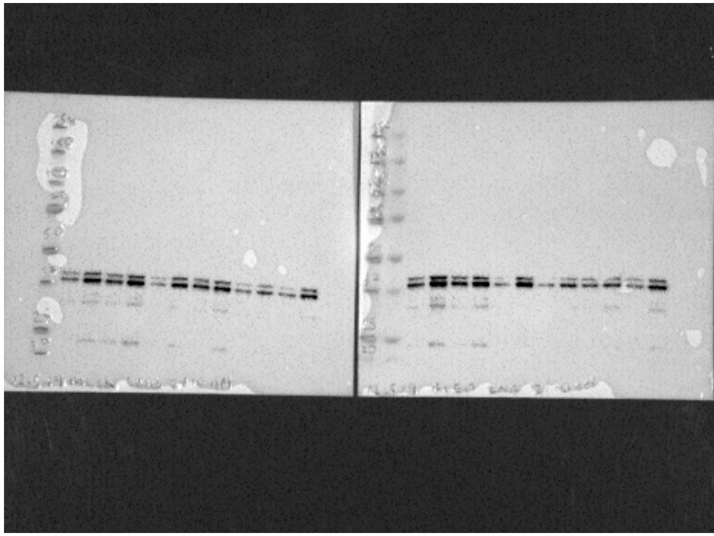

Exposure 5 sec

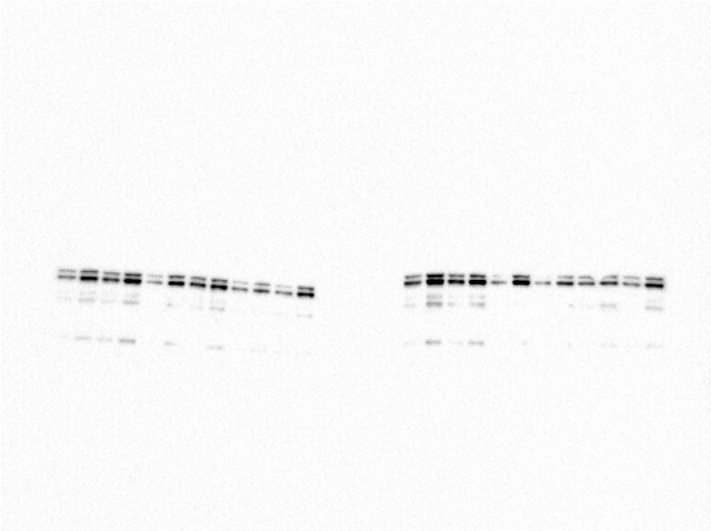

Figure 1A Actin

Exposure 50 sec

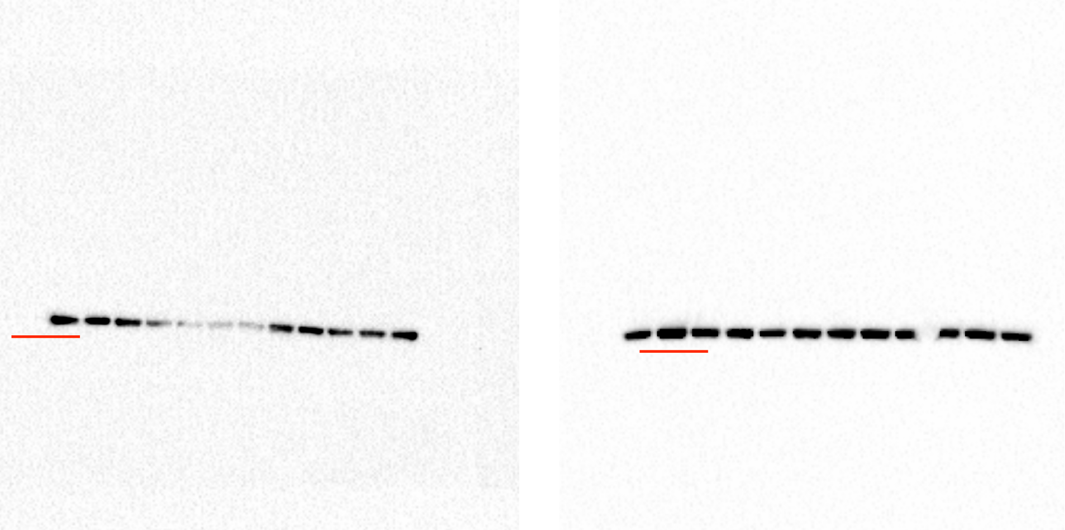

Exposure 10 sec

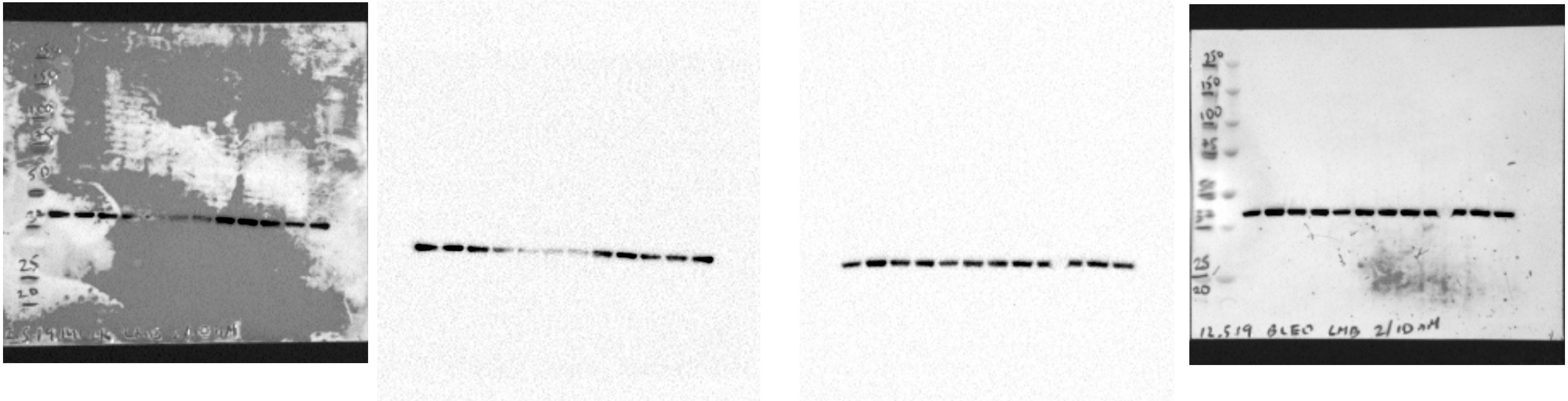

Figure 1B pEGFR

Exp 1200 sec

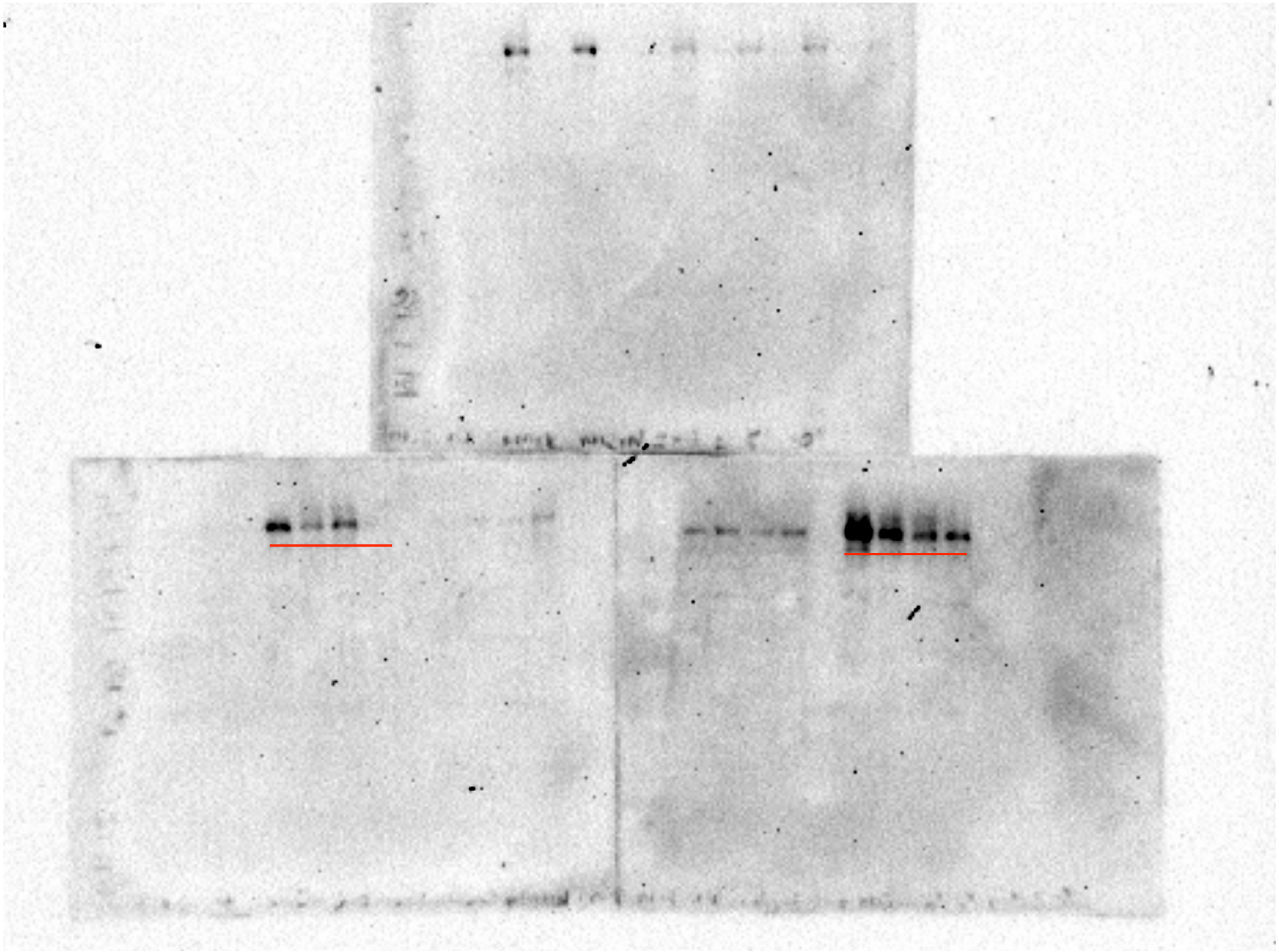

Figure 1B pEGFR

Exp 480 sec

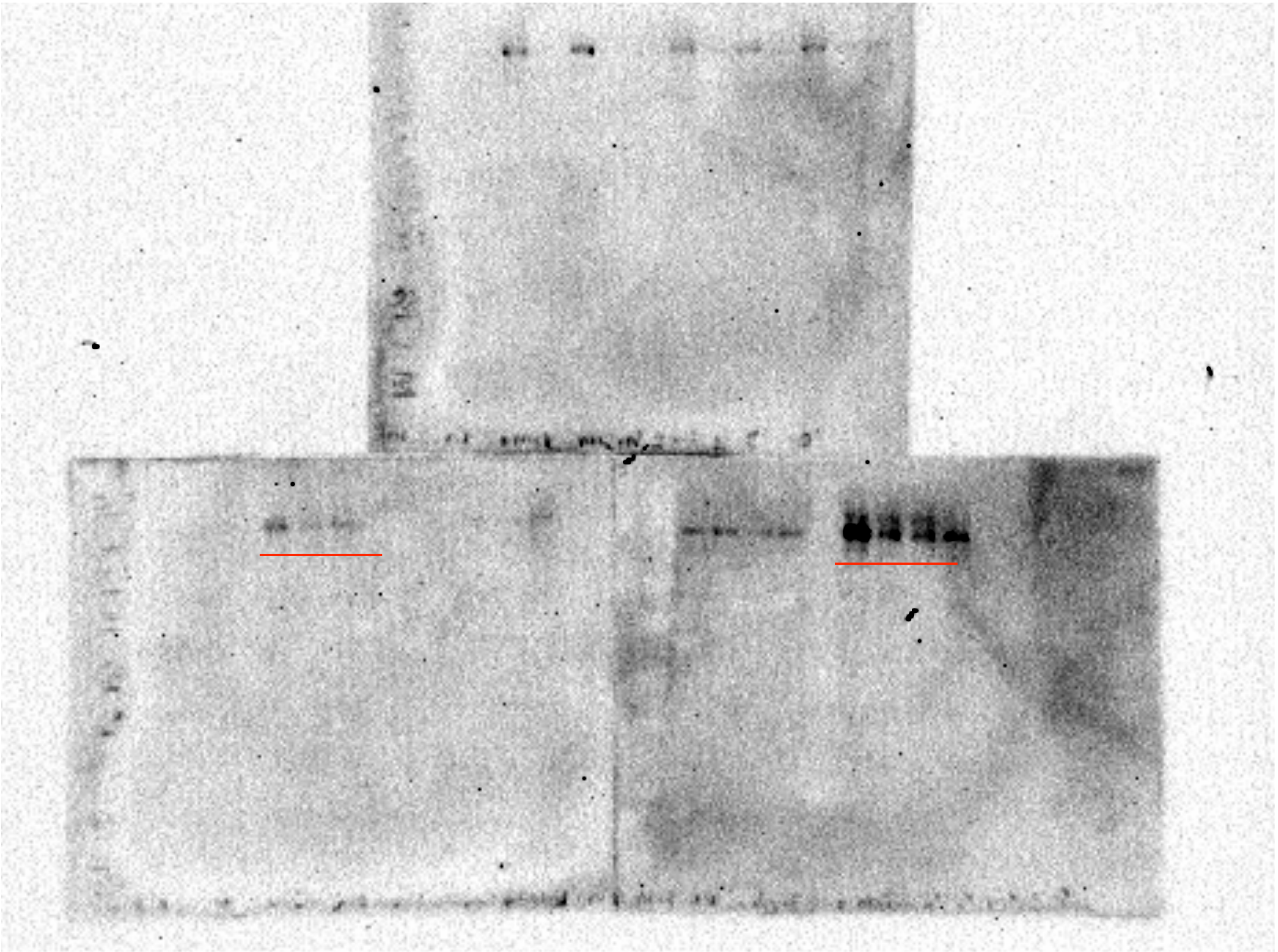

Figure 1B Actin

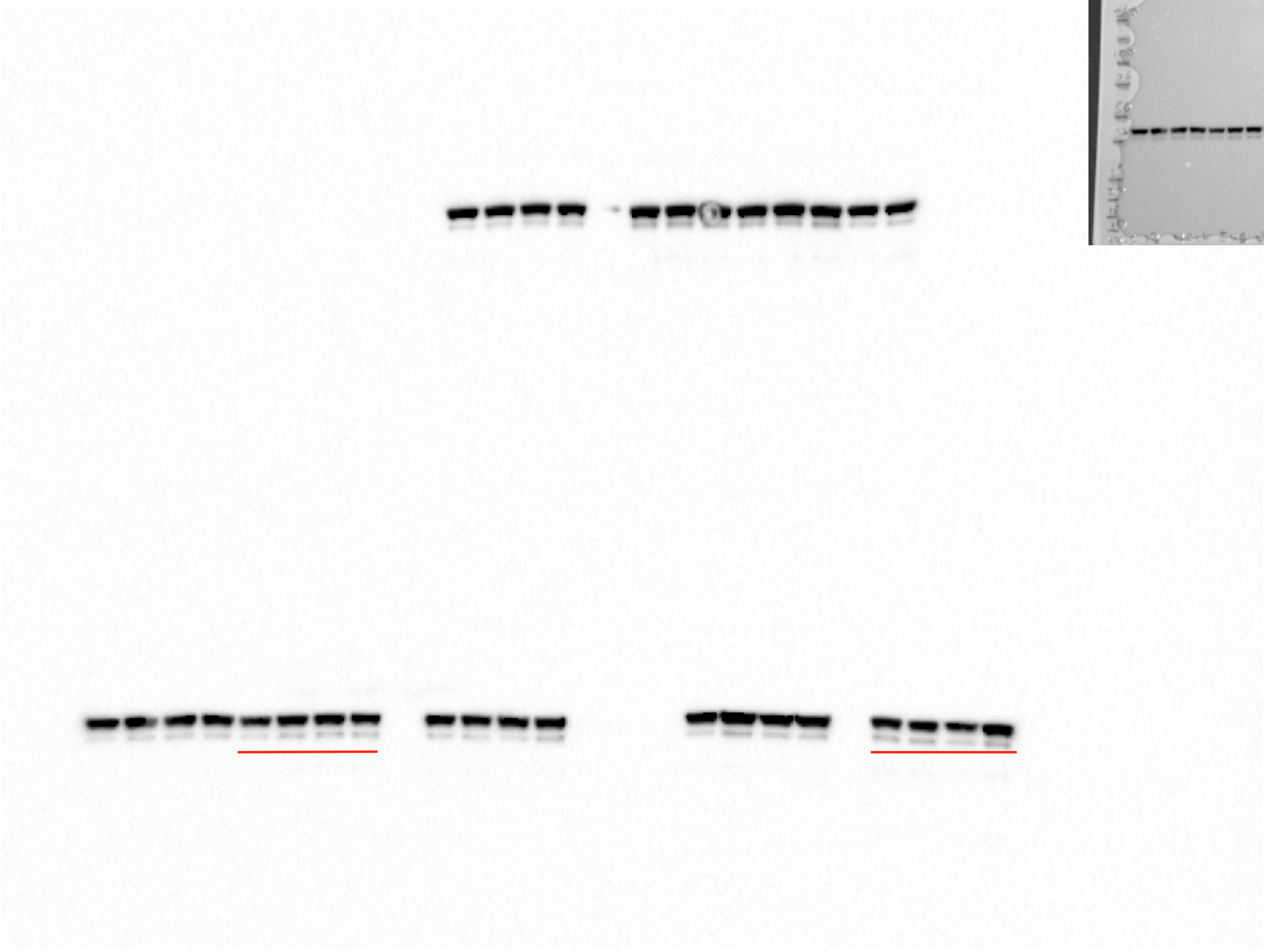

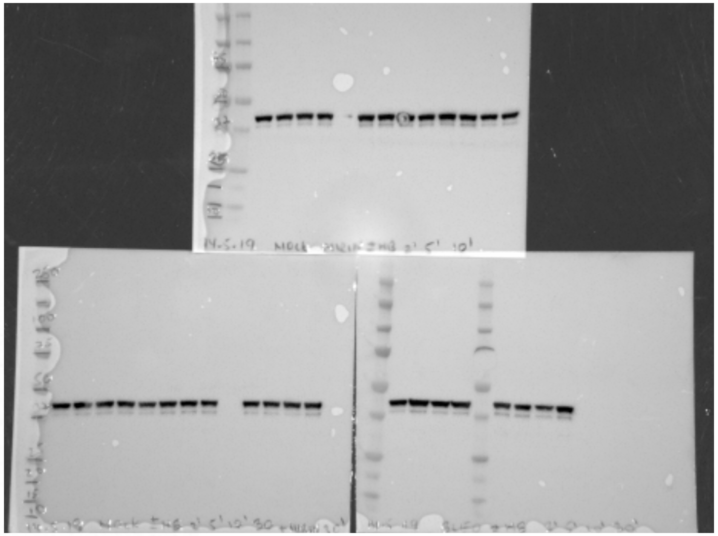

Figure 1D pEGFR

Exp 50 sec

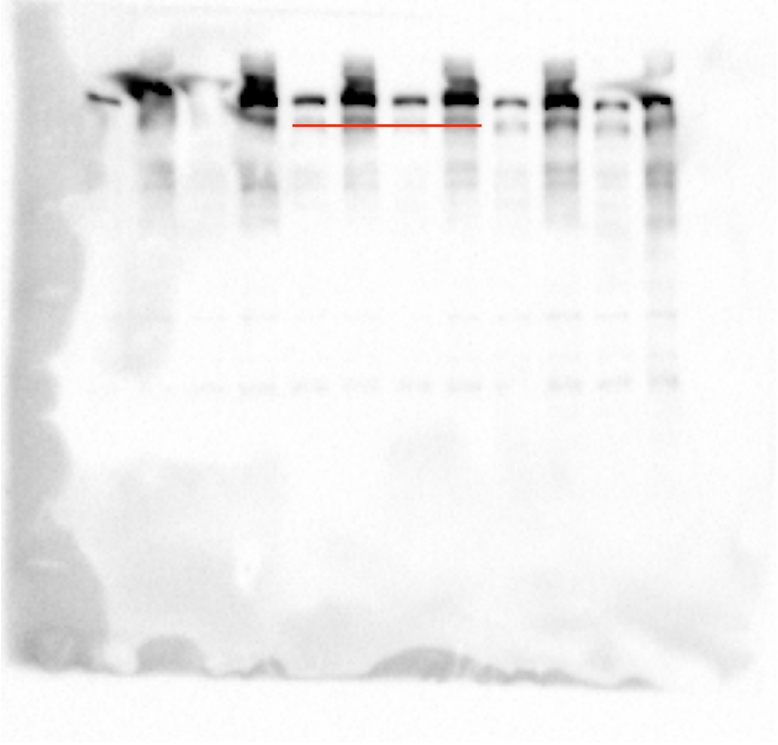

Exp 10 sec

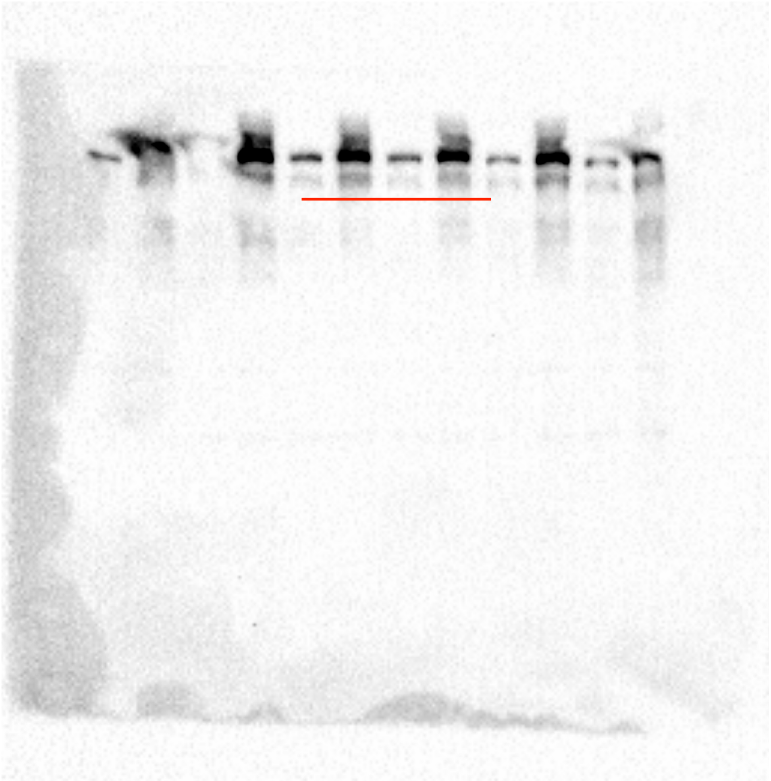

Figure 1D pAKT

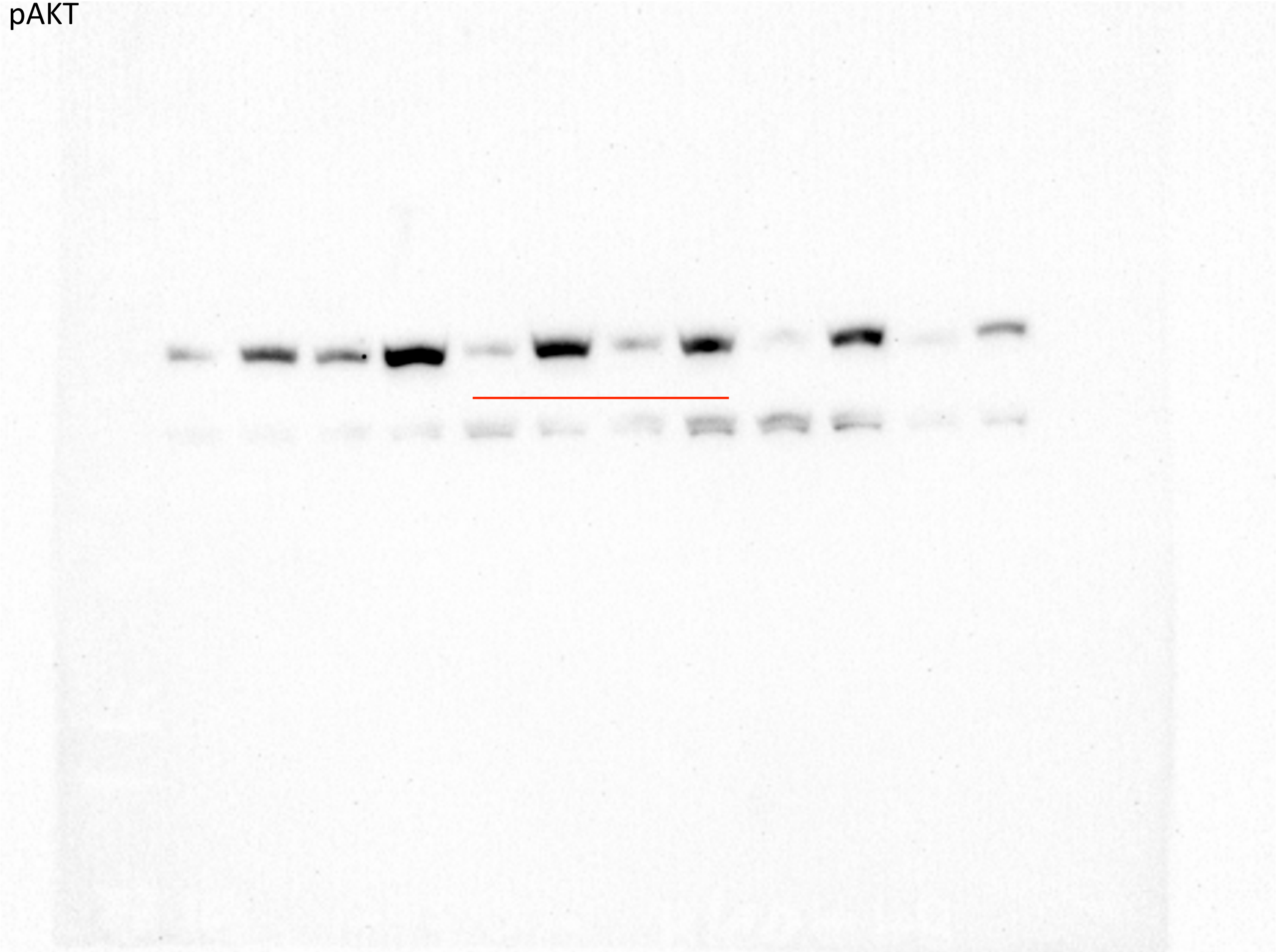

Figure 1D pERK

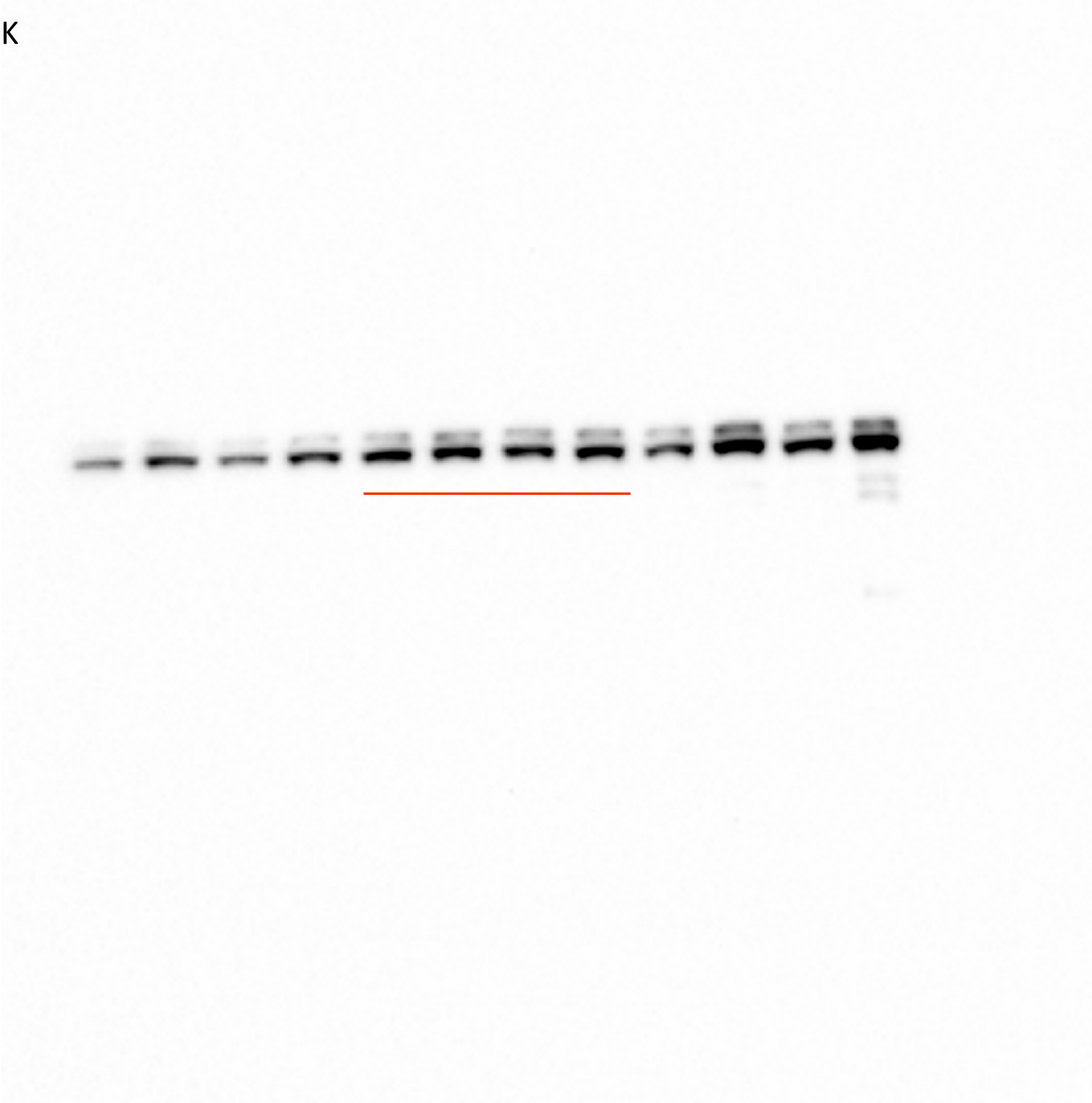

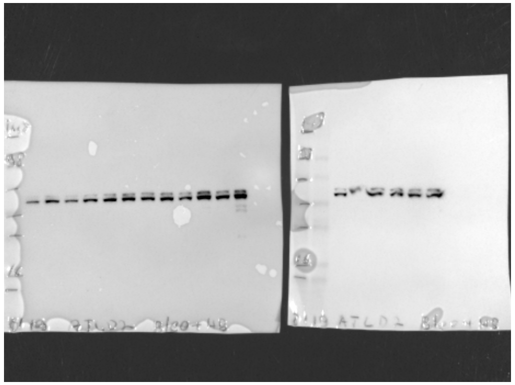

Figure 1D Actin

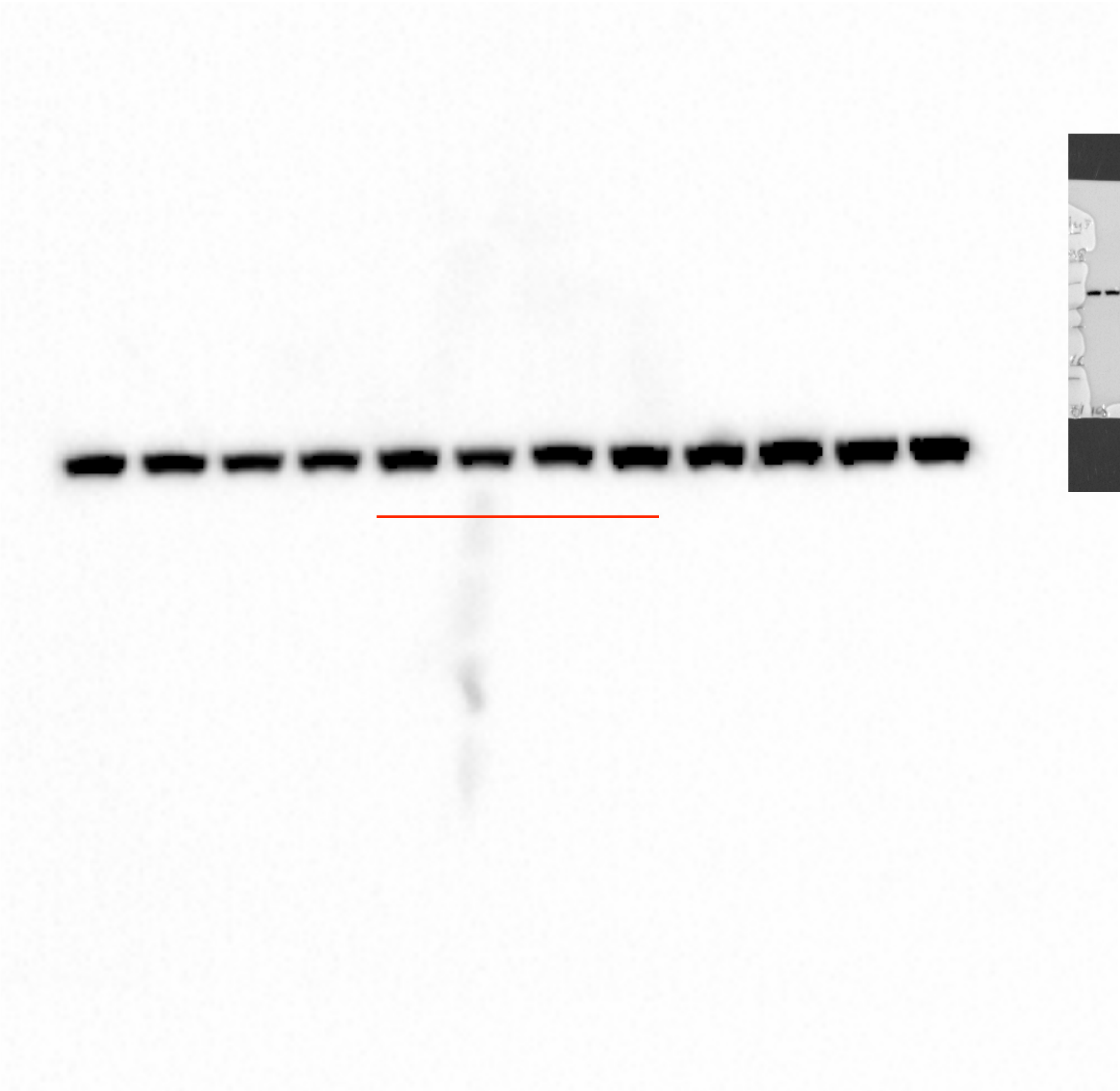

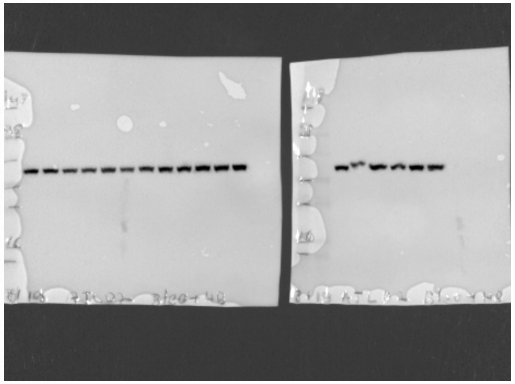

Figure S1B pEGFR

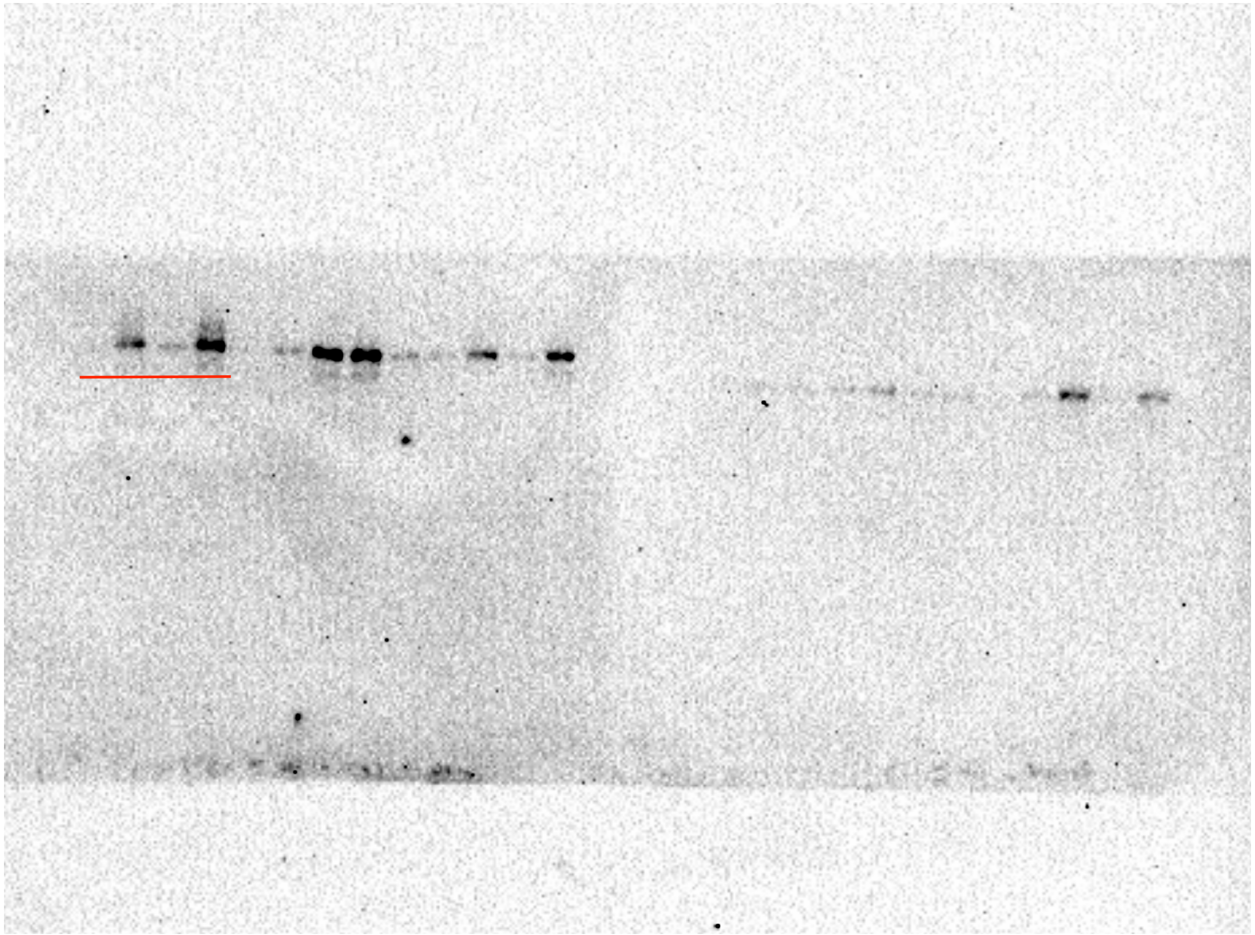

Figure S1B Actin

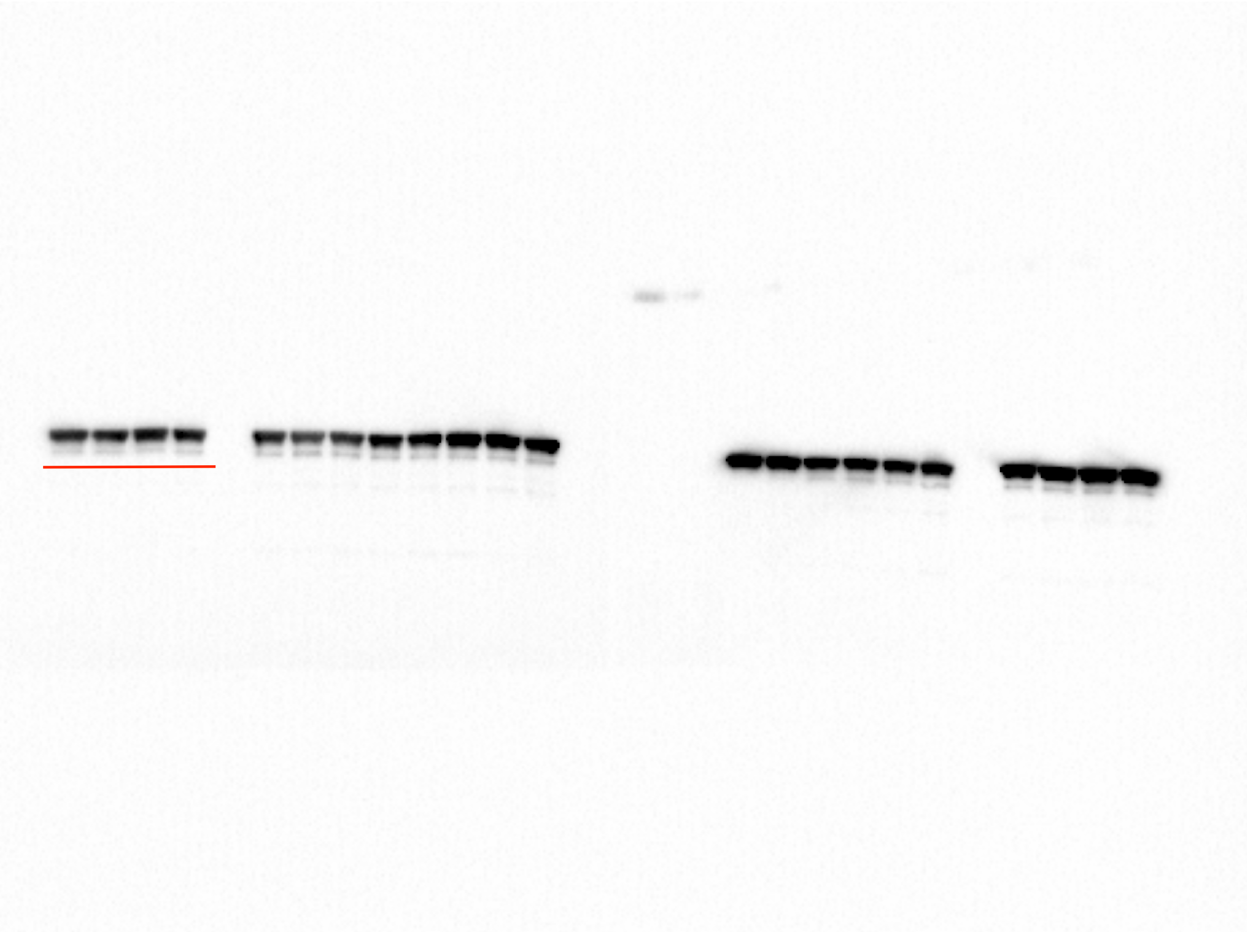

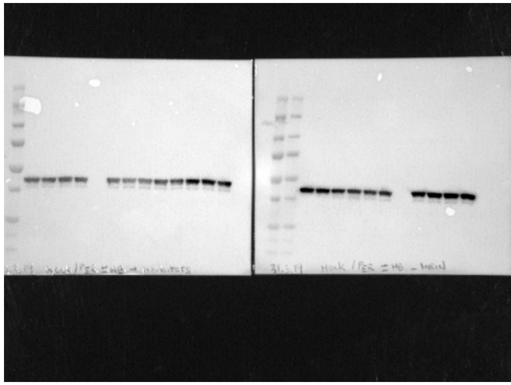

Figure 2A pEGFR

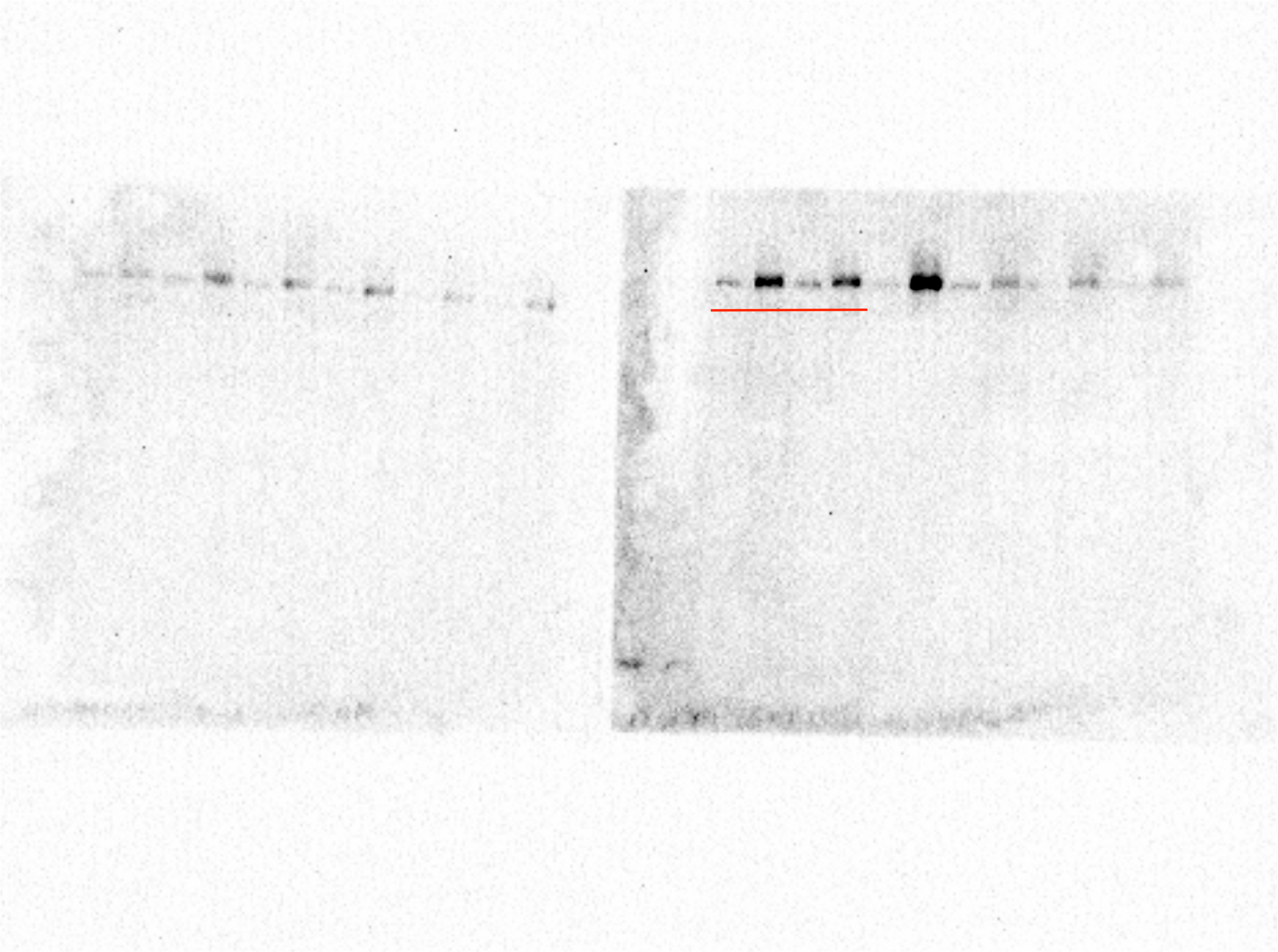

Figure 2A pERK

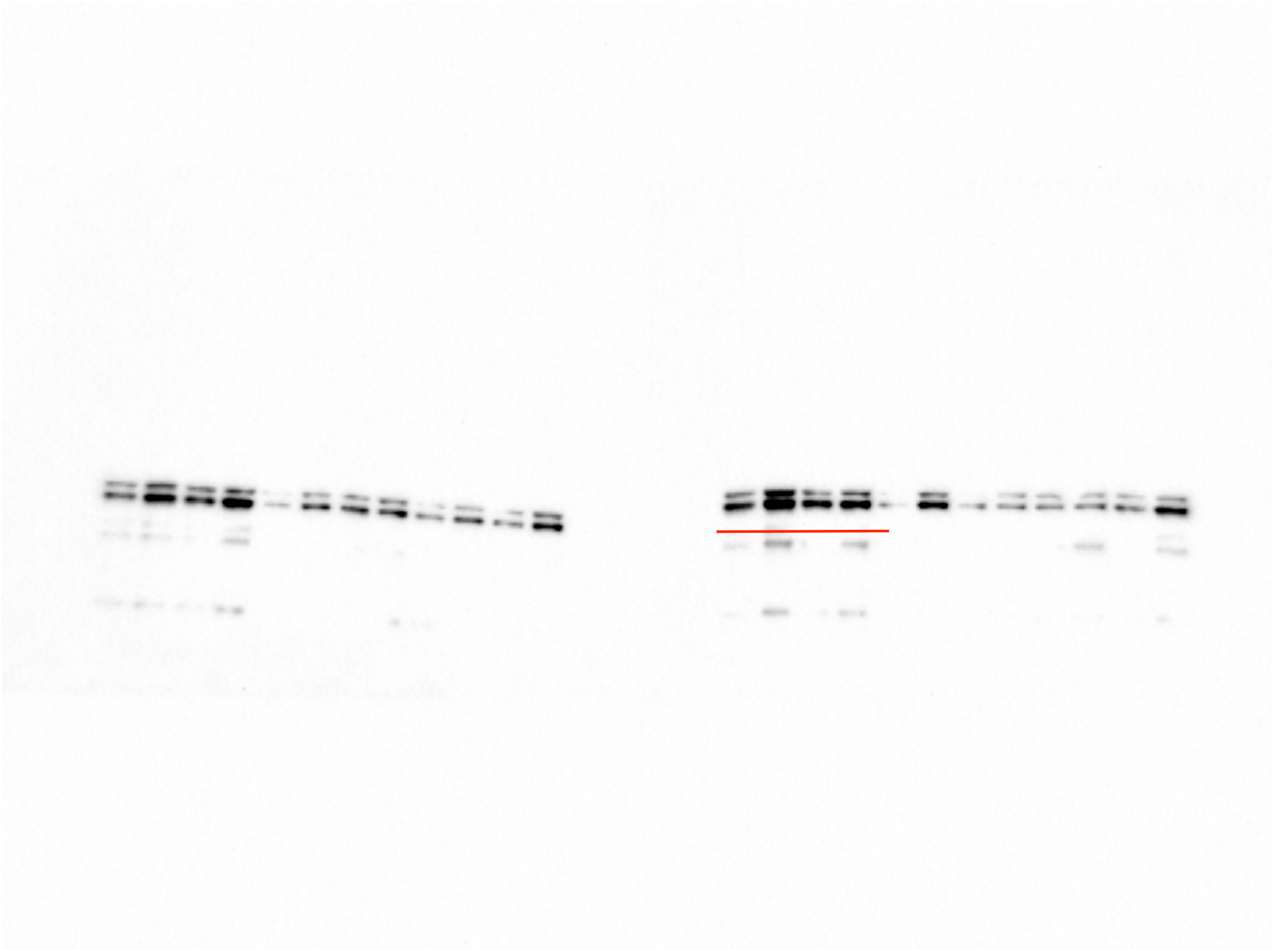

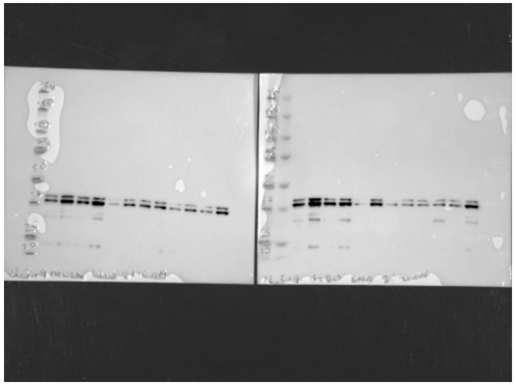

Figure 2A Actin

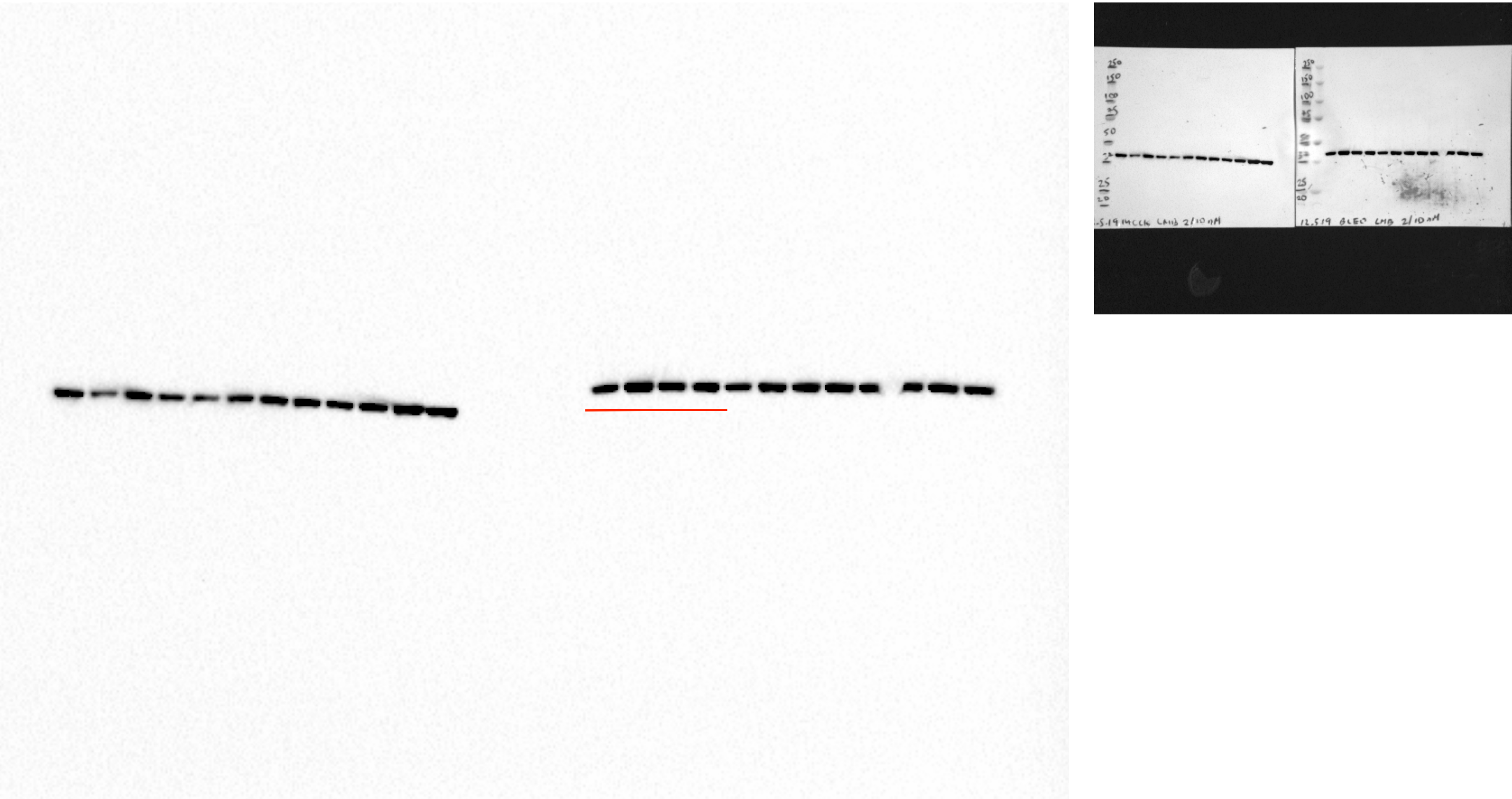

Figure 2B pEGFR mut

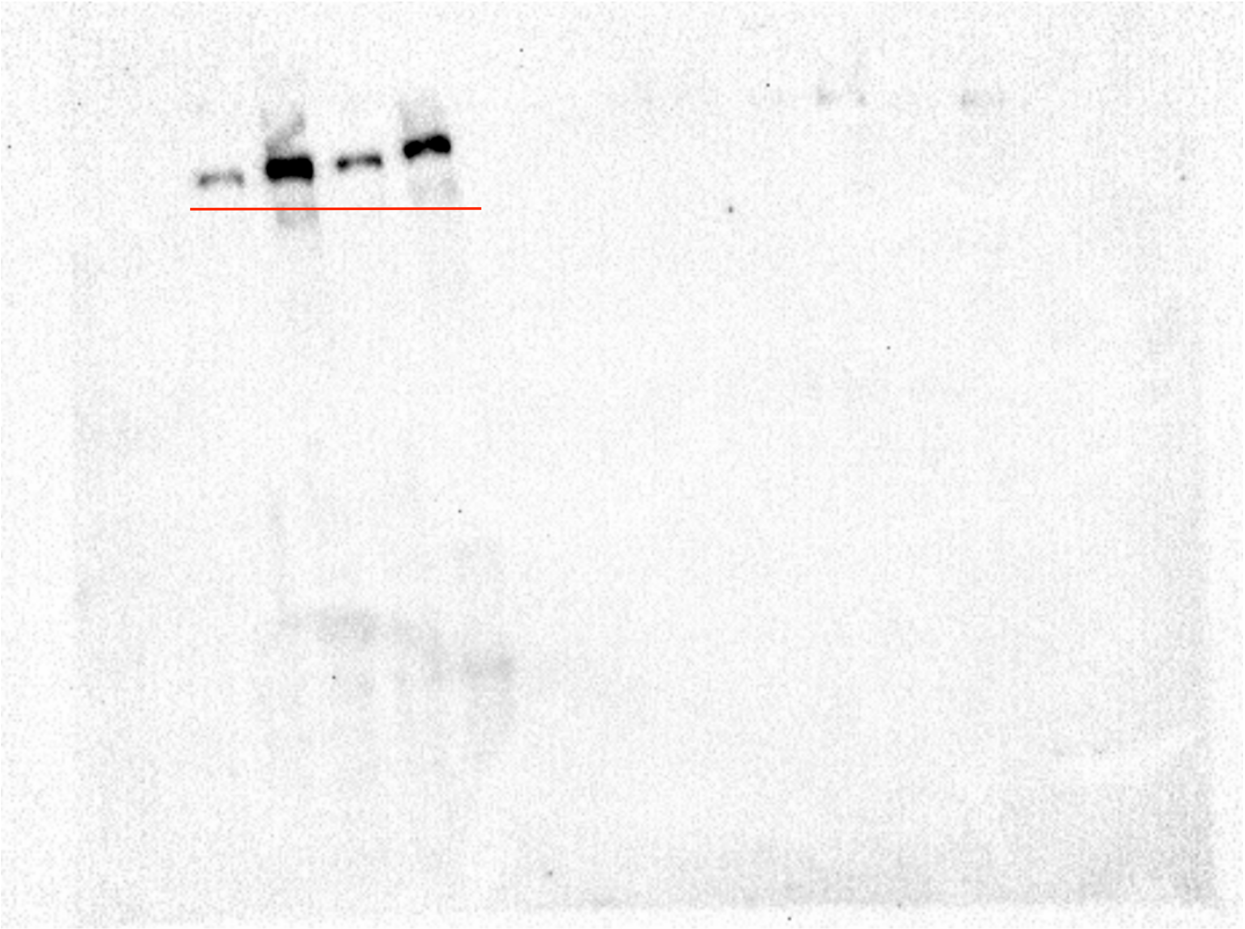

Figure 2B pERK mut

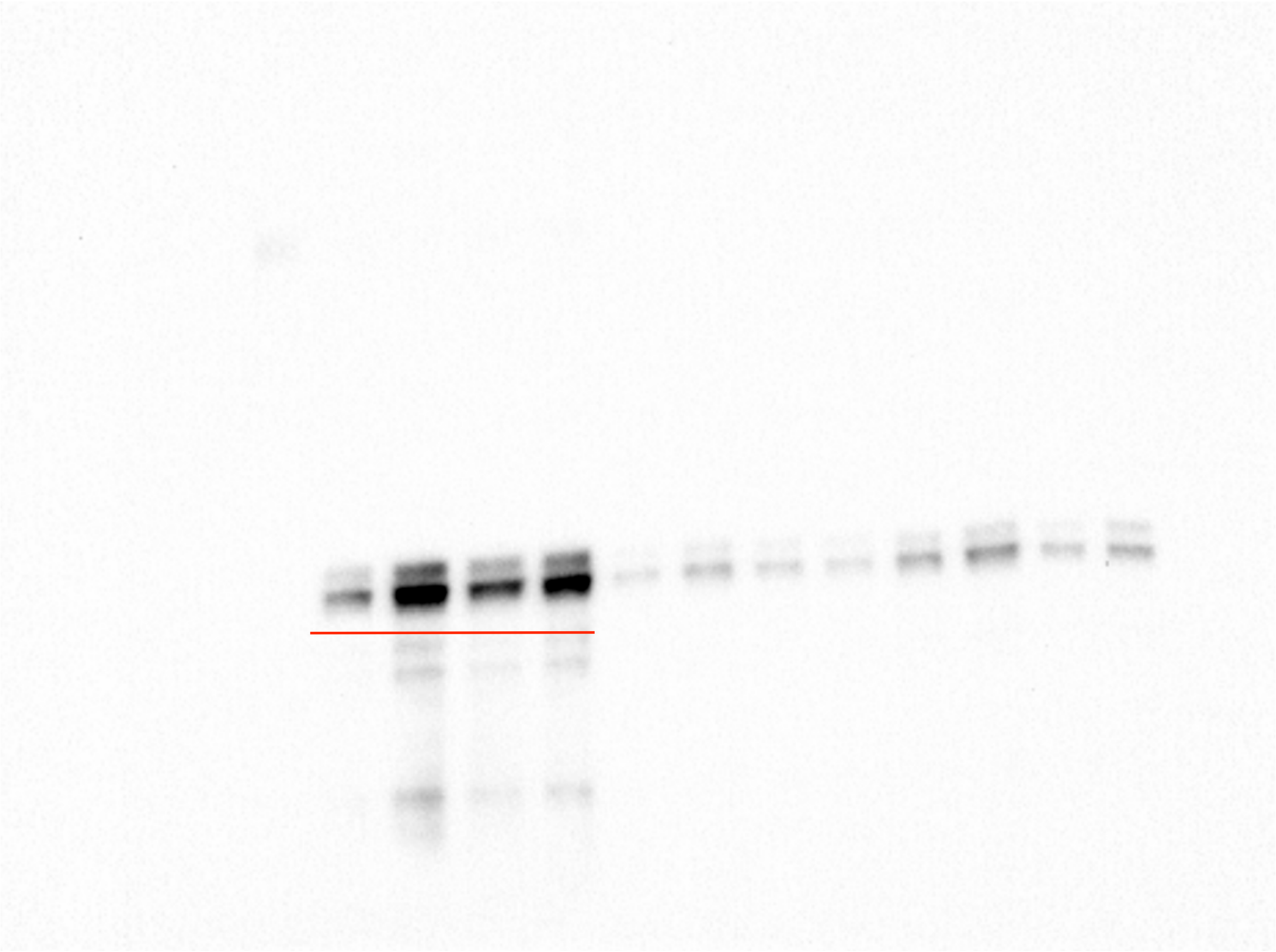

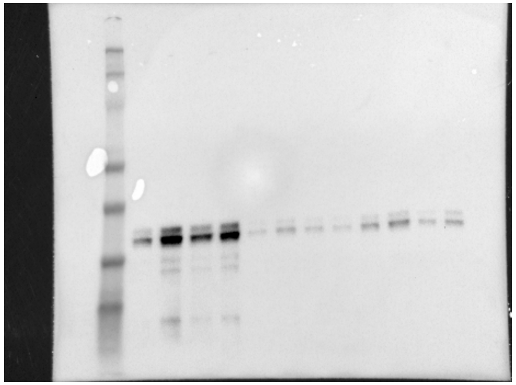

Figure 2B Actin mut

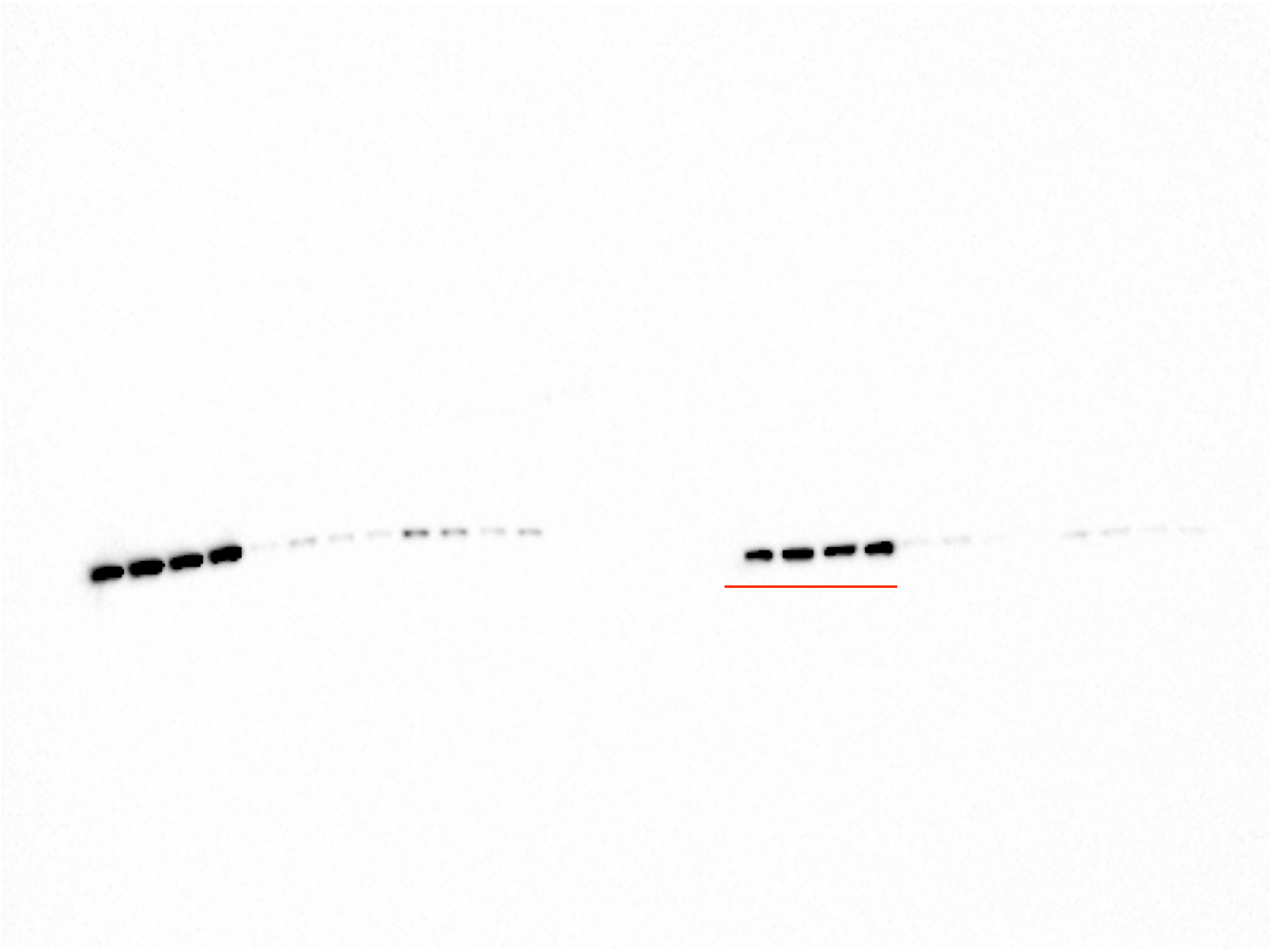

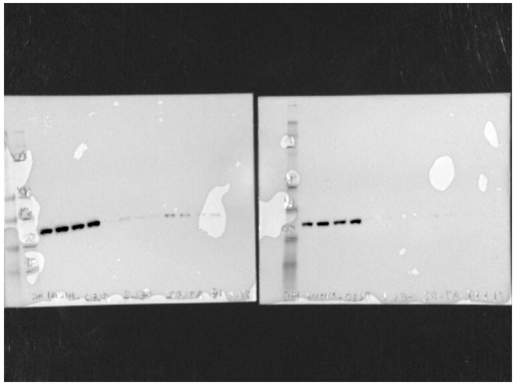

Figure 2B pEGFR WT

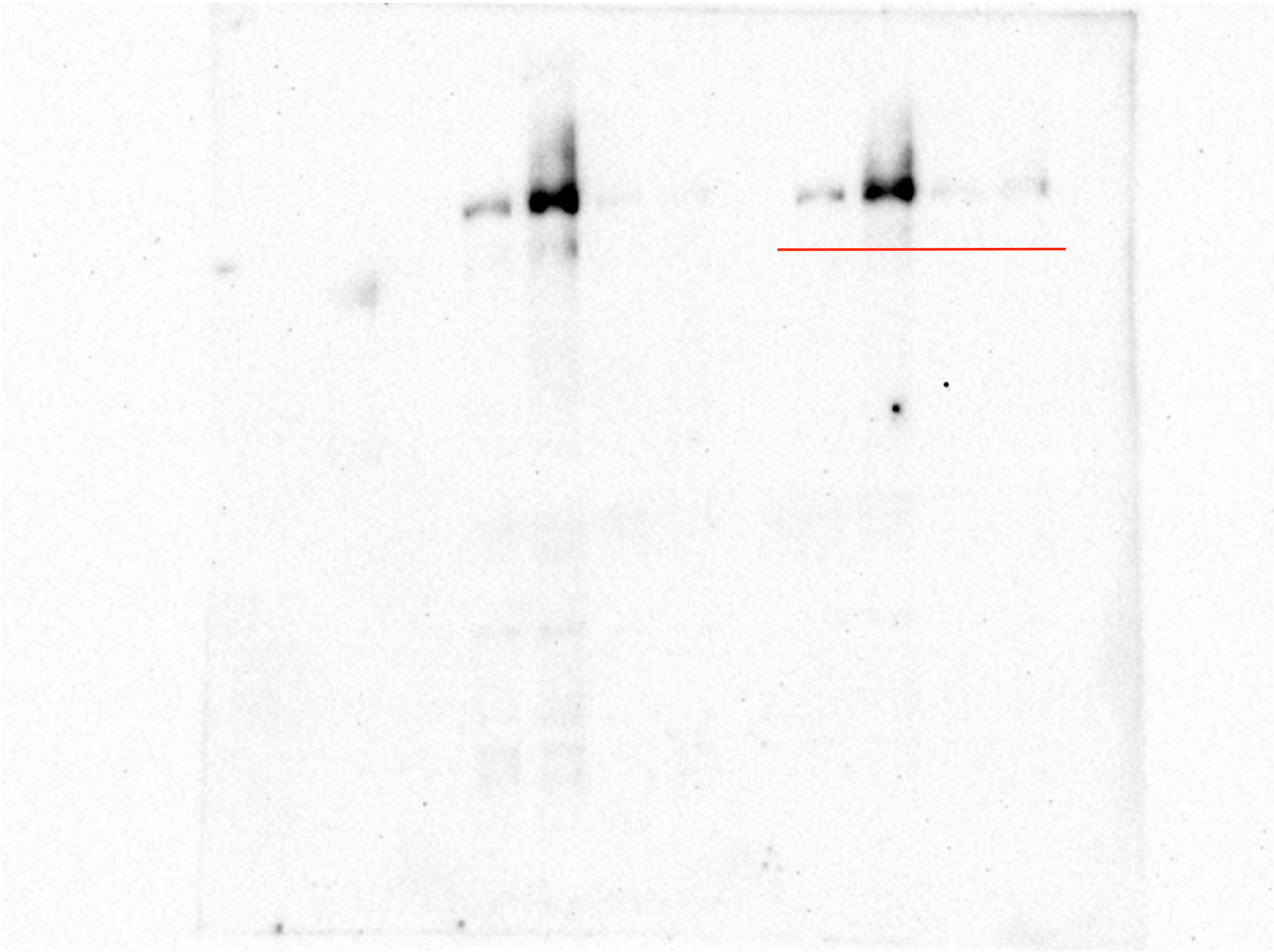

Figure 2B pERK WT

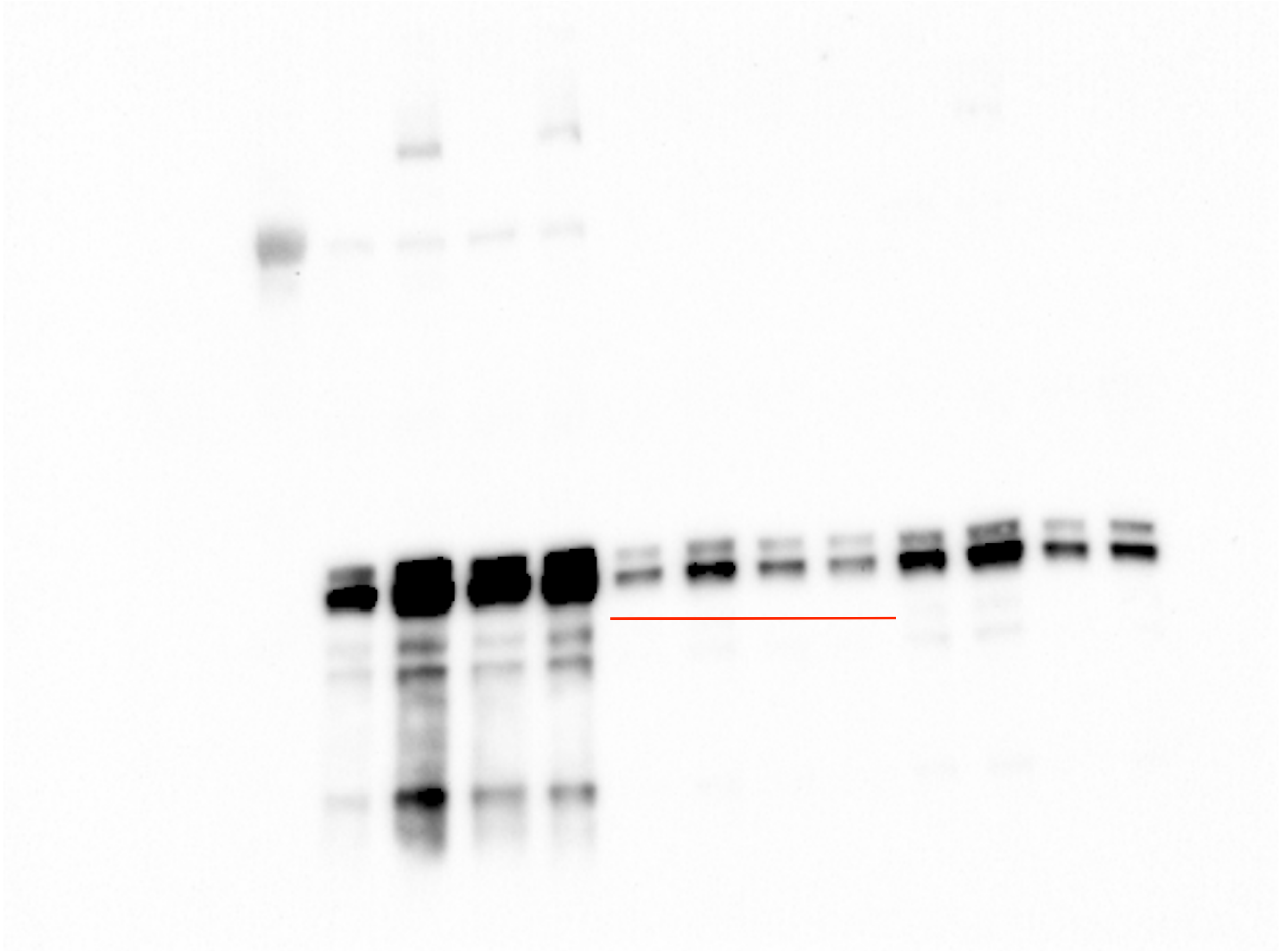

Figure 2B Actin WT

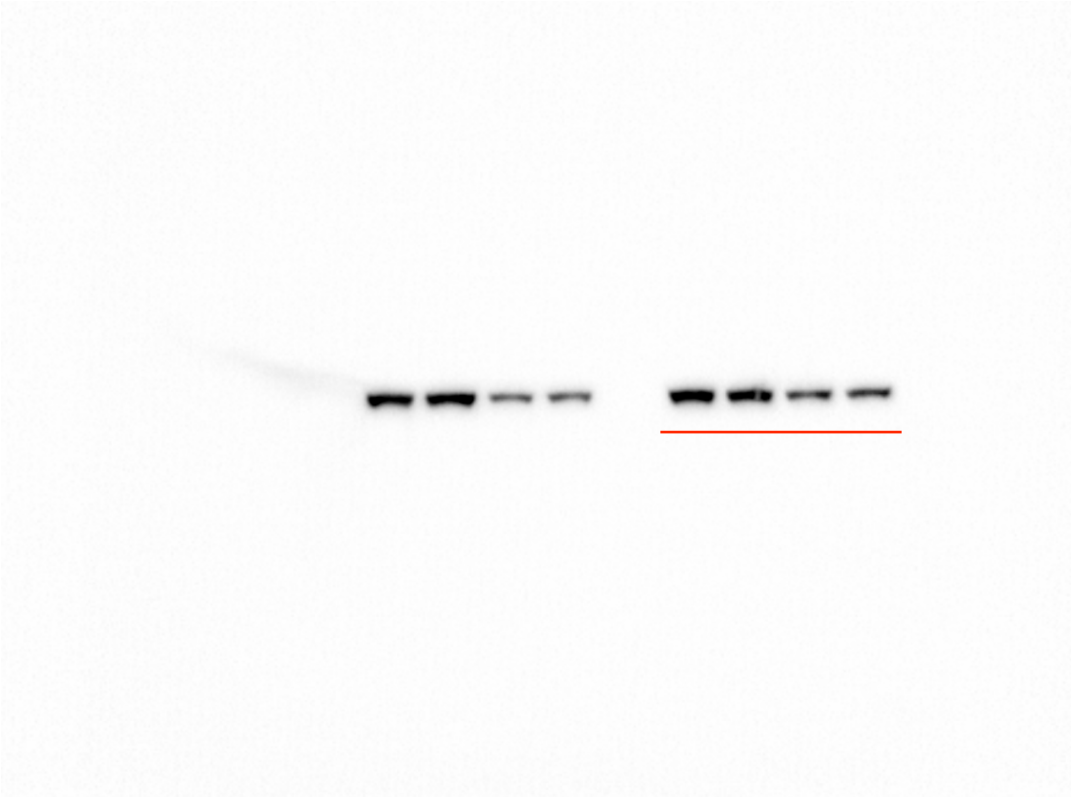

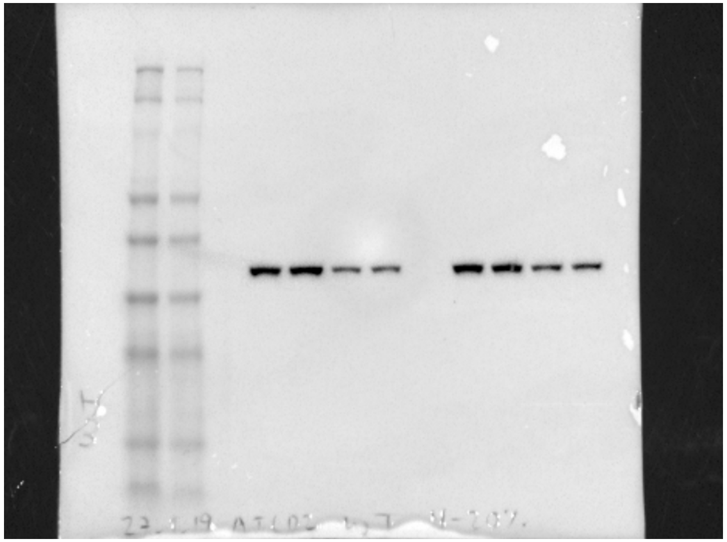

Figure 2C pEGFR

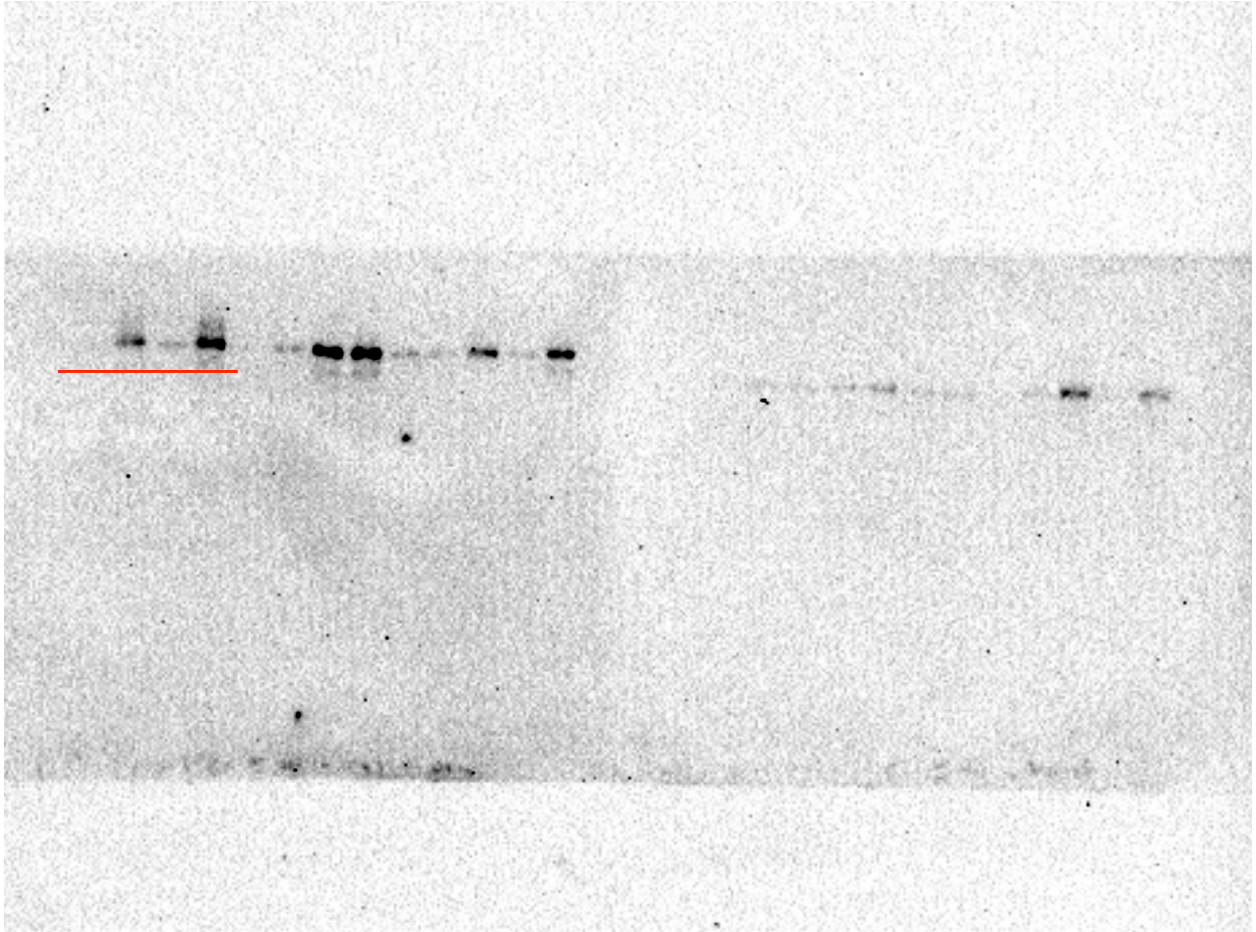

Figure 2C pAKT

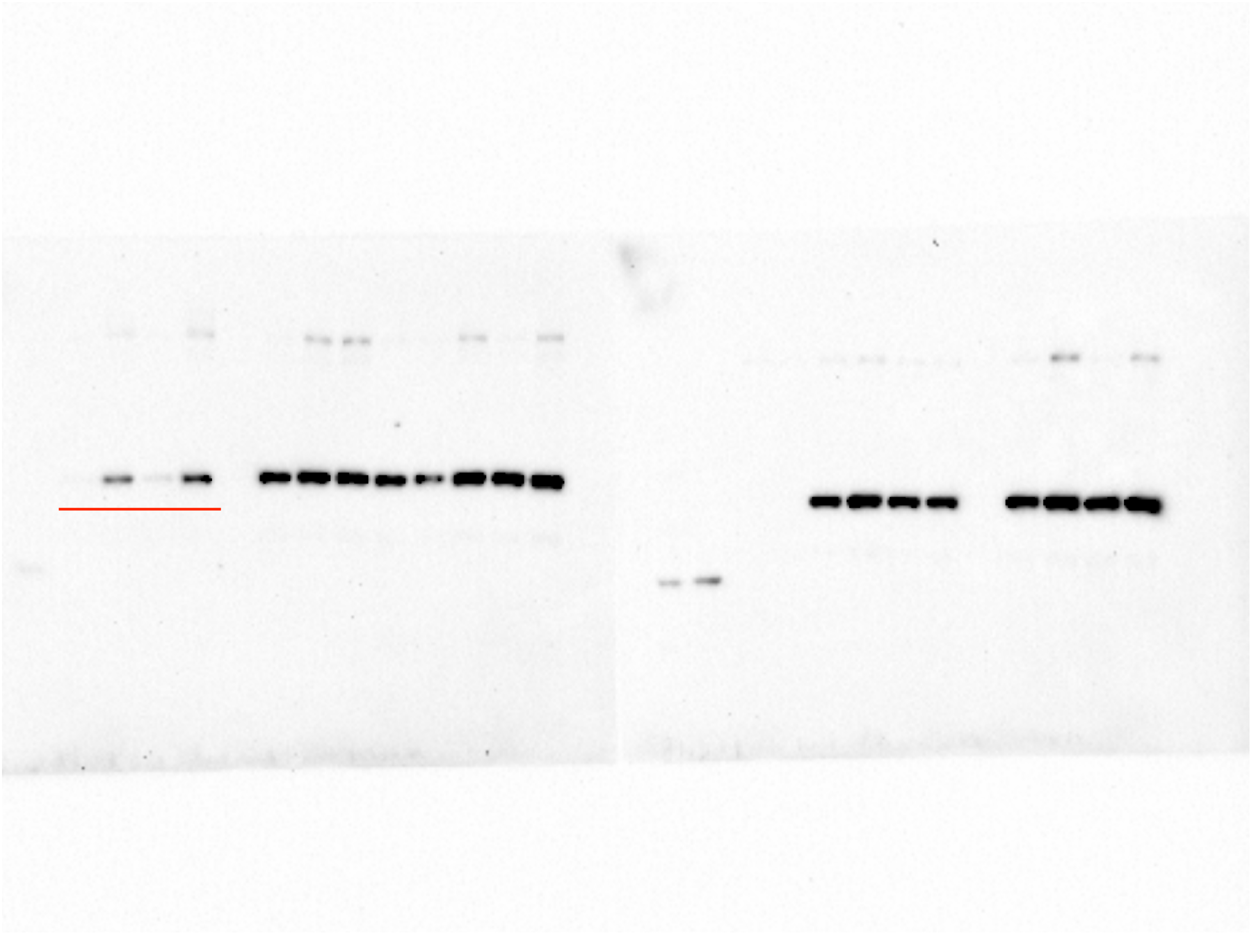

Figure 2C pERK

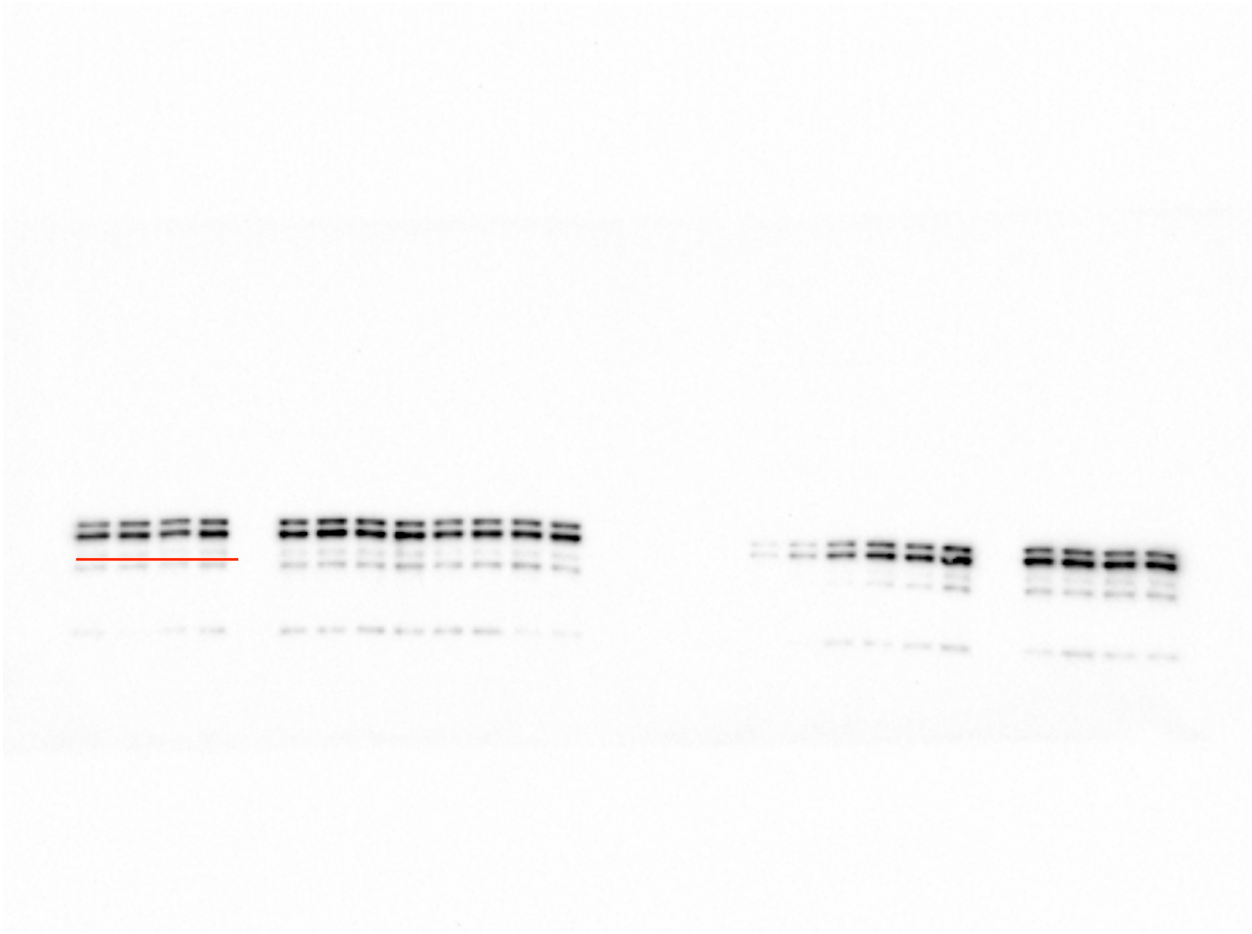

Figure 2C Actin

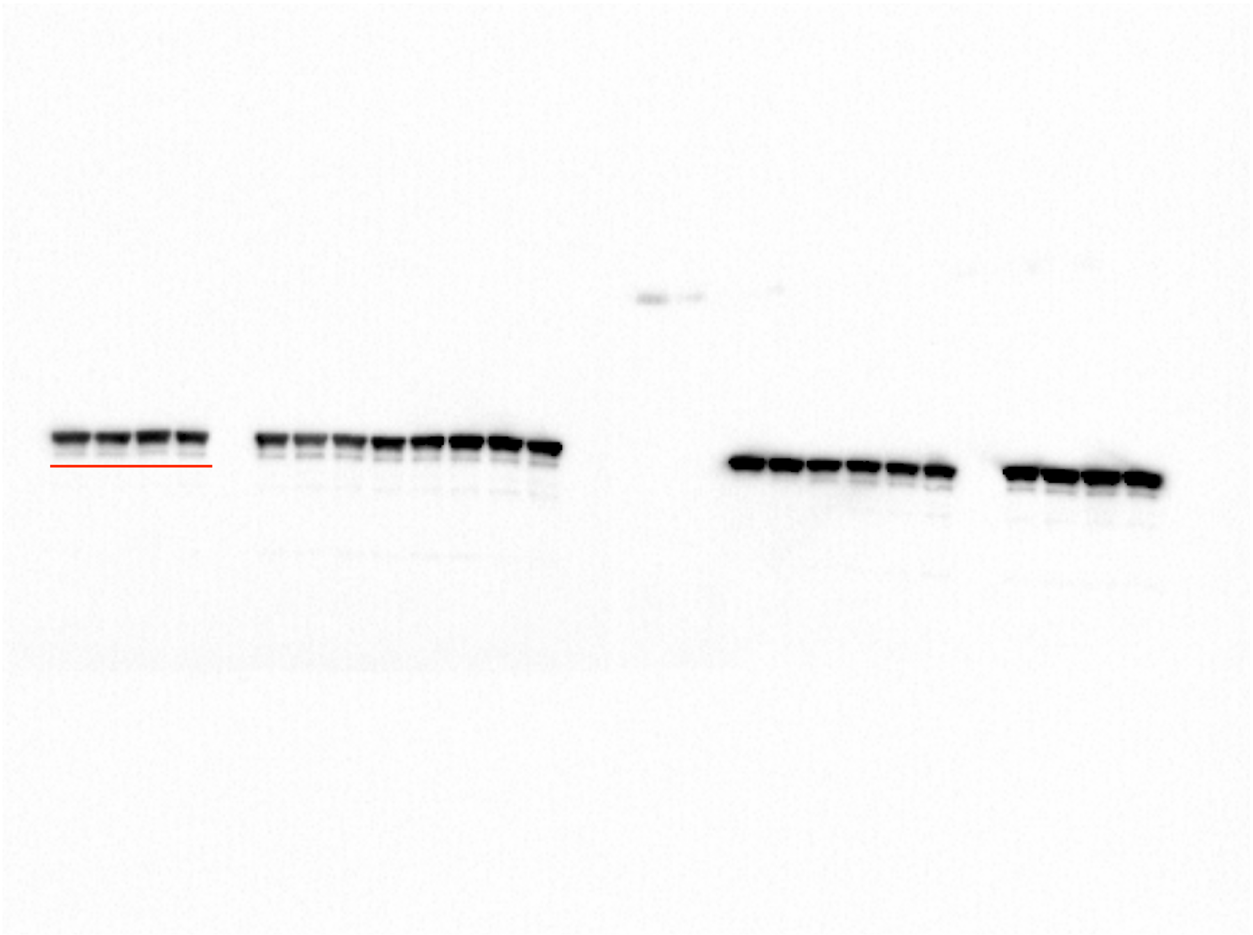

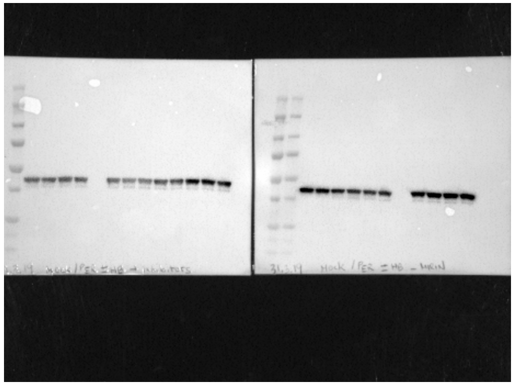

Figure S2A pEGFR

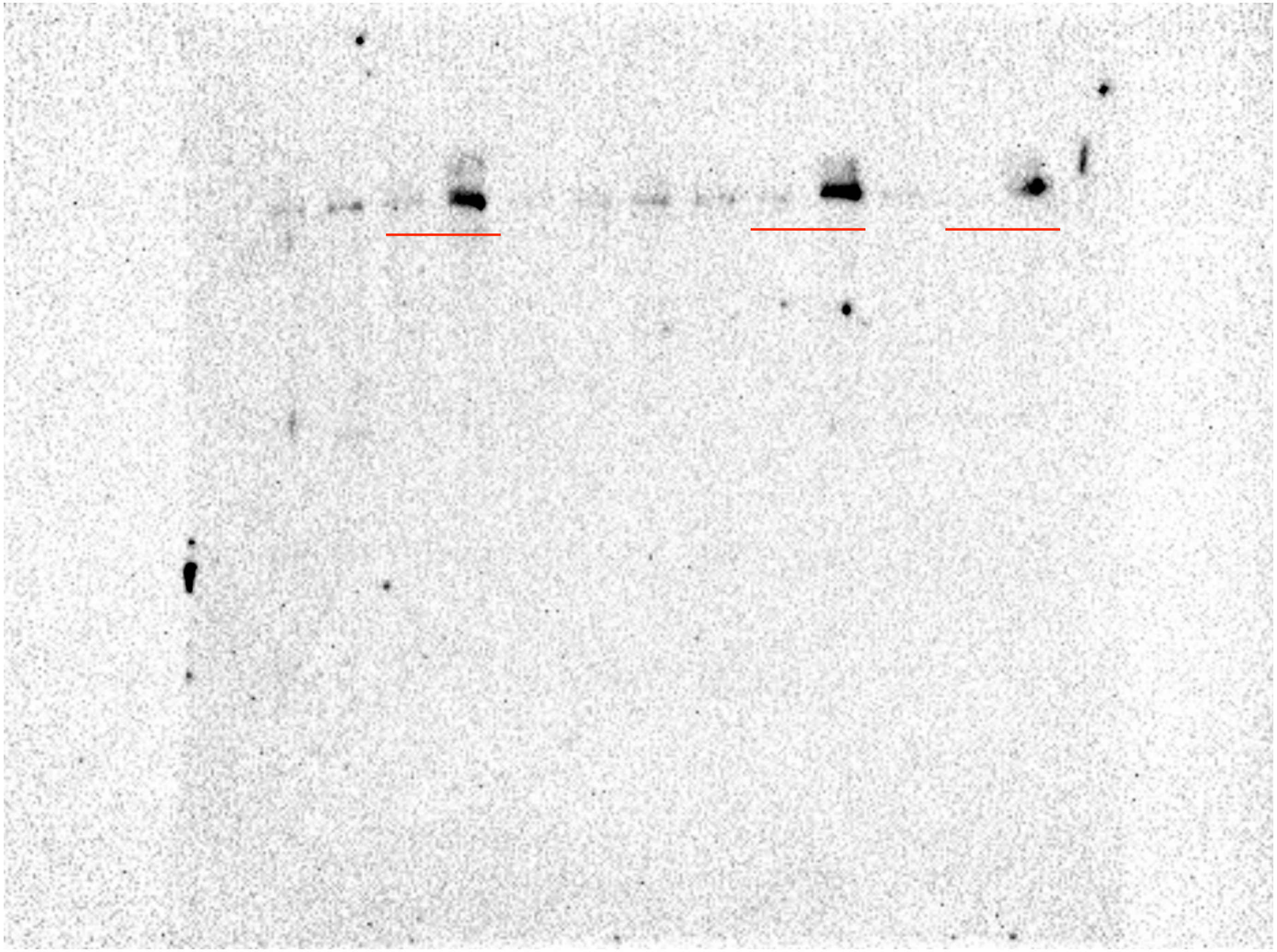

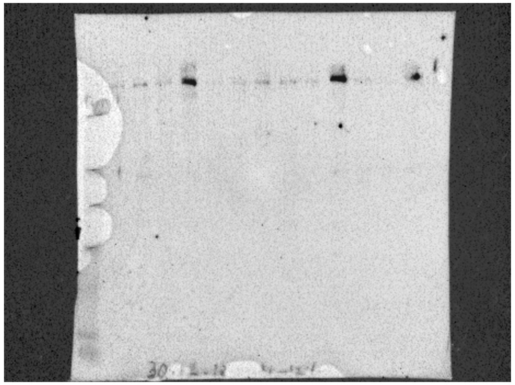

Figure S2A pERK

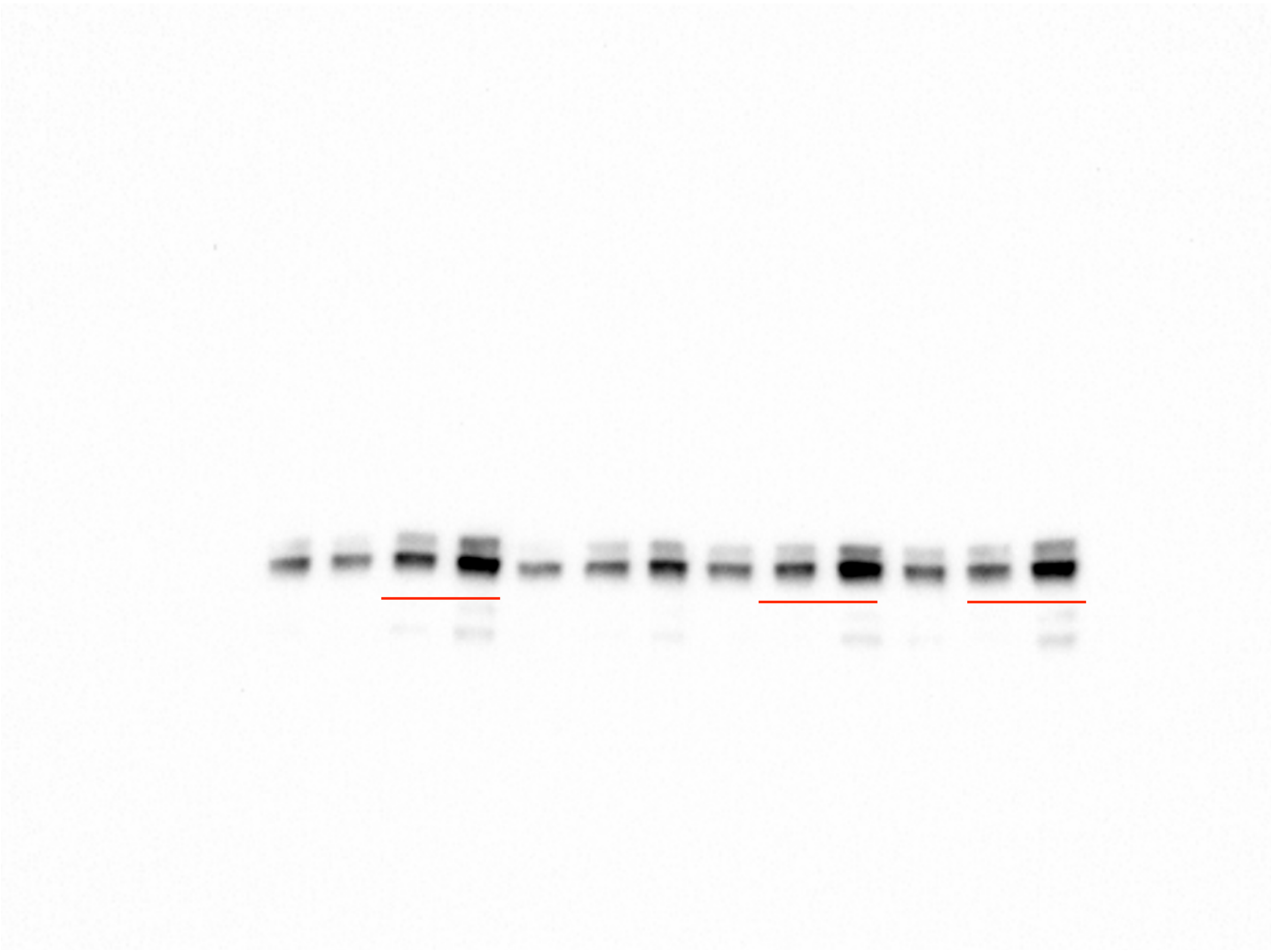

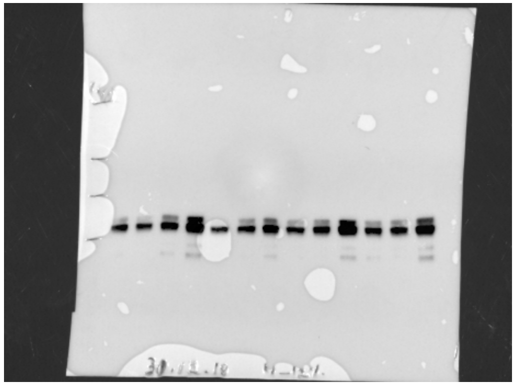

Figure S2A Actin

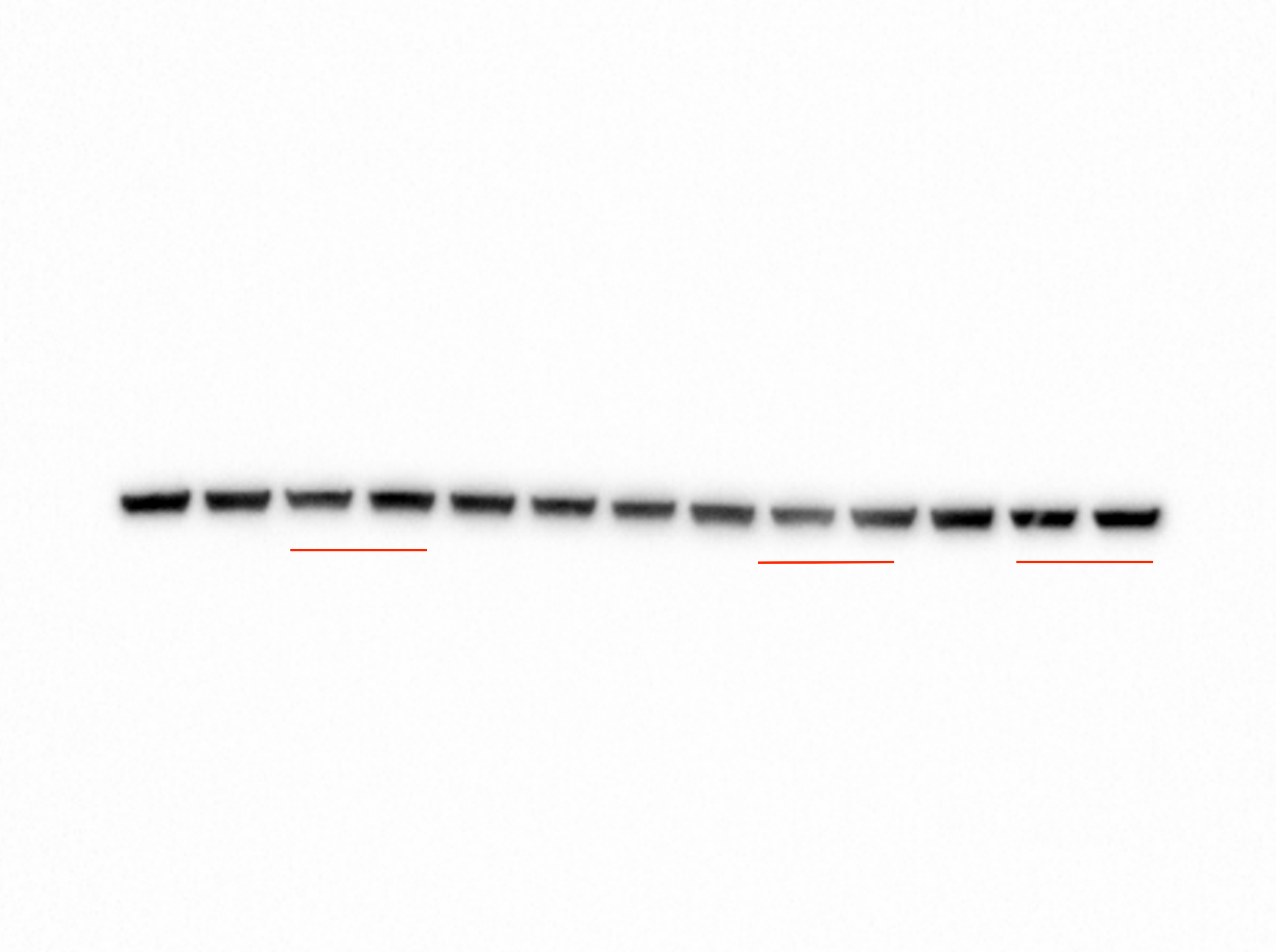

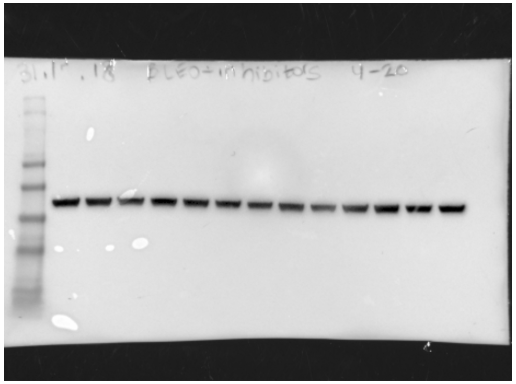

Figure S2B pEGFR

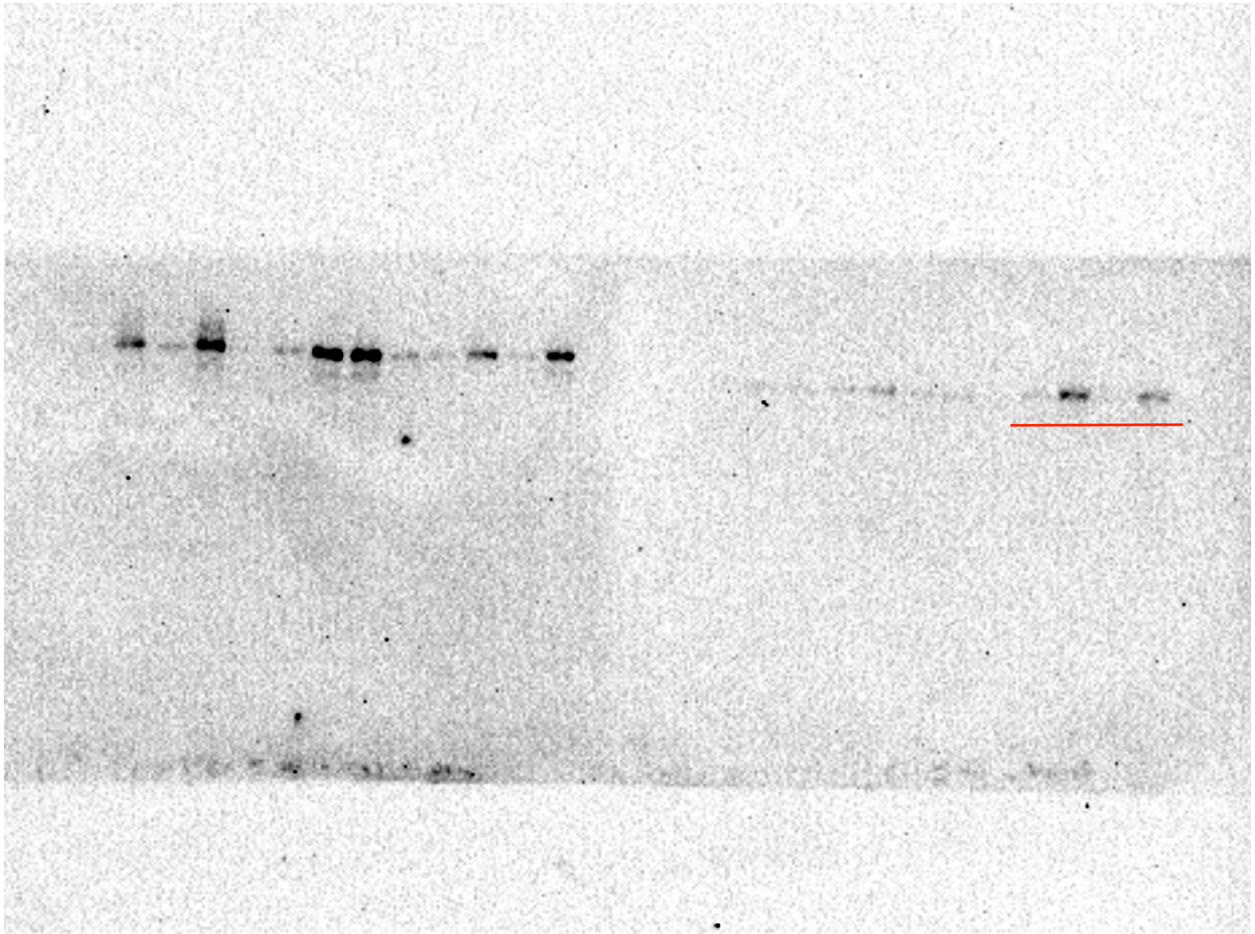

Figure S2B Actin

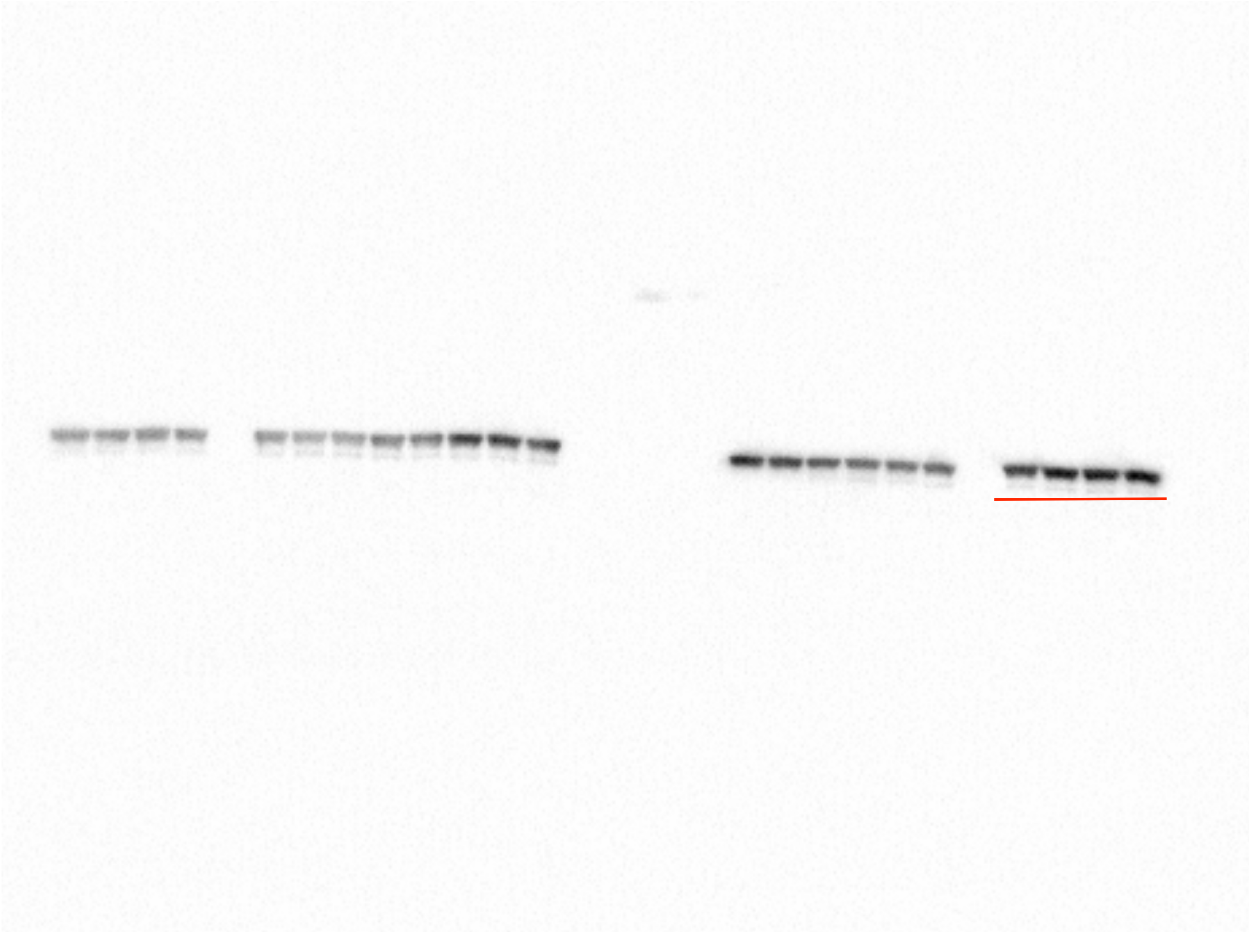

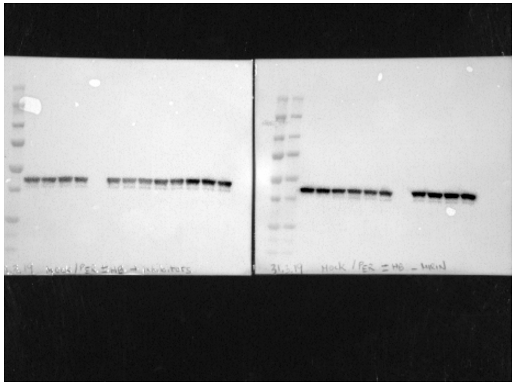

Figure S2C pEGFR

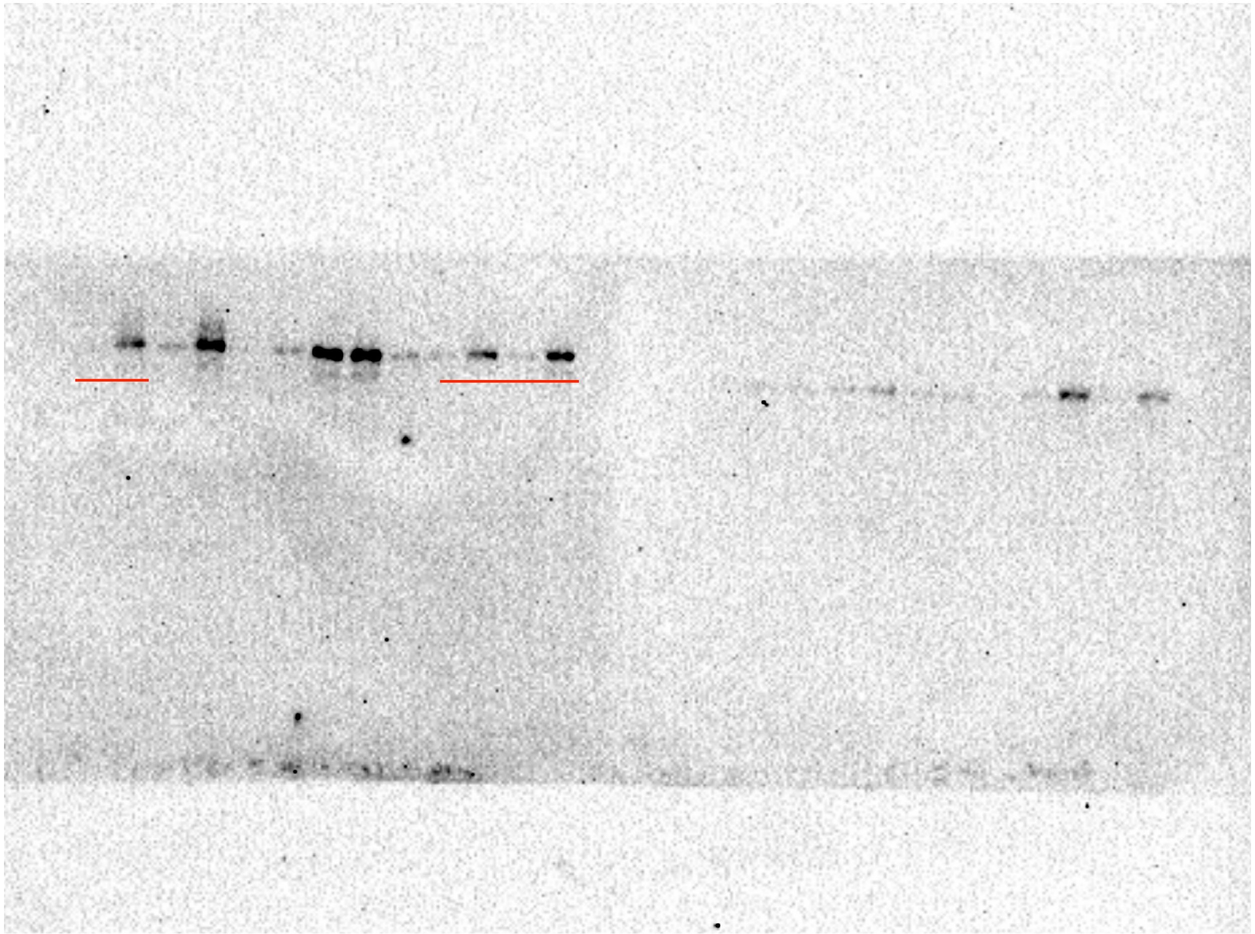

Figure S2C Actin

Exp 10 sec

Exp 1 sec

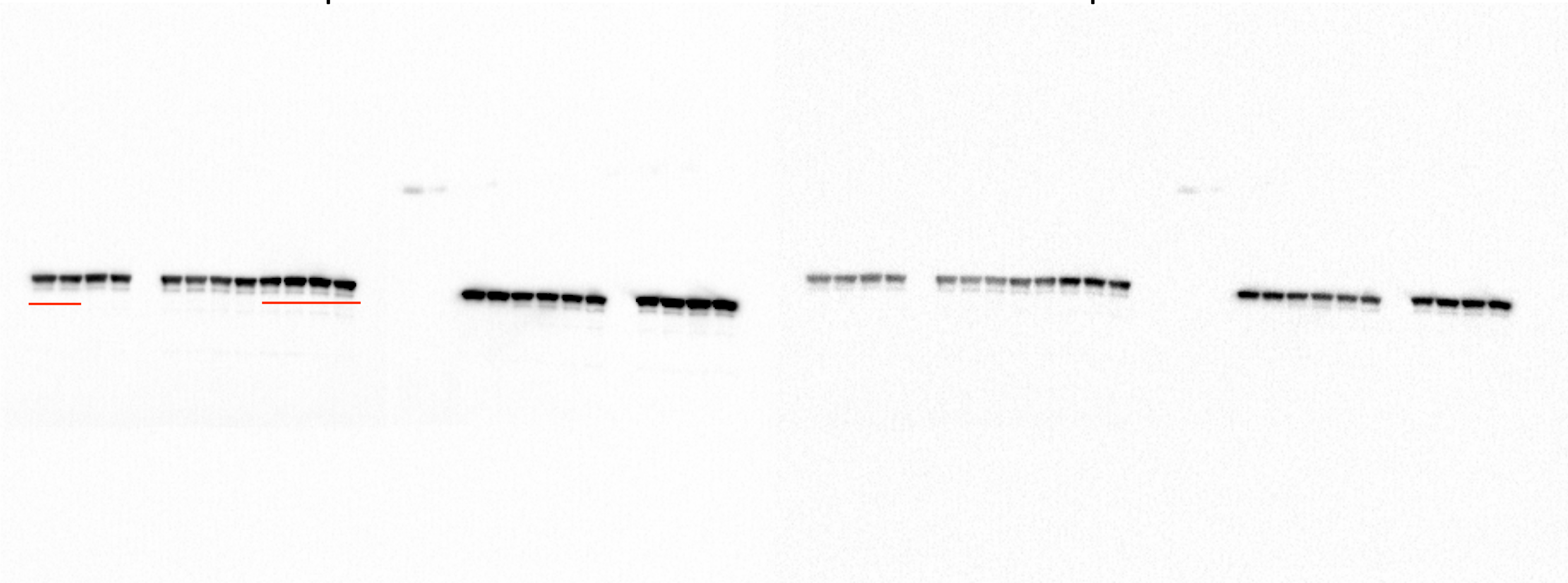

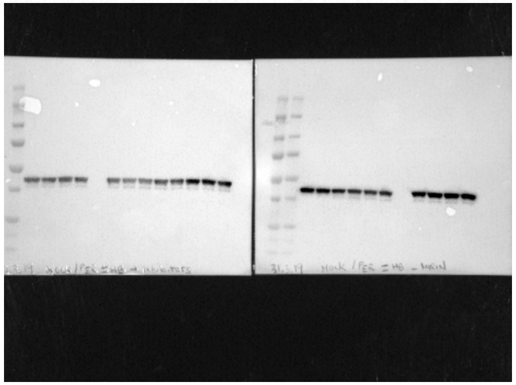

Supplement: Supplementary file 2 — Supplementary Information 2. [file 41598_2022_9779_MOESM2_ESM.pdf]
